# *Irf7* Deficiency Confers Protection Against Influenza Infection, Independent of *irf3*

**DOI:** 10.7150/ijbs.126714

**Published:** 2026-01-22

**Authors:** Jianzhou Cui, Sherman S. W. Foo, Wan Ting Kong, Chenshi Lin, Patrick B. Ampomah, Olga Zharkova, Loo Soon Chai, Karishma Sachaphibulkij, Suruchi Arora, Nivashini Kaliaperumal, Hong Meng Lim, John Connolly, Anna-Marie Fairhurst, Jinmiao Chen, Lina H. K. Lim

**Affiliations:** 1 Immunology Translational Research Program, Yong Loo Lin School of Medicine, National University of Singapore, Singapore, Singapore.; 2 Department of Physiology, Yong Loo Lin School of Medicine, National University of Singapore, Singapore, Singapore.; 3 Immunology Program, Life Sciences Institute, National University of Singapore, Singapore, Singapore.; 4 NUS-Cambridge Immunophenotyping centre, National University of Singapore, Singapore, Singapore.; 5 Institute of Molecular and Cellular Biology (IMCB), Agency for Science, Technology and Research, Singapore, Singapore.; 6 Singapore Immunology Network (SIgN), Agency for Science, Technology and Research, Singapore, Singapore.; 7 Bioinformatics Institute (BII), Agency for Science, Technology and Research, Singapore, Singapore.; 8 Center for Computational Biology and Program in Cancer and Stem Cell Biology, Duke-NUS Medical School, Singapore, Singapore.

**Keywords:** *irf3*, * irf7*, macrophages, DCs, single cell sequencing, influenza

## Abstract

Interferon regulatory factors *irf3* and *irf7* are pivotal for antiviral immunity, yet their cell-type-specific contributions, particularly within macrophage and dendritic cell (DC) lineages, have not been fully elucidated. Here, employing a multi-omics strategy encompassing *in vitro* assays, *in vivo* influenza A virus (IAV) infection models, NanoString, transcriptomic analyses, and scGPT-based computational modeling, we dissect the divergent and context-dependent roles of *irf3* and *irf7*. We demonstrate macrophages exhibit heightened sensitivity to TLR3 stimulation, a response critically dependent on *irf3*. Conversely, DCs respond more robustly to TLR7 activation and very weakly to TLR3 activation. Unexpectedly, global *Irf7*^-/-^ mice displayed enhanced survival against IAV-induced lethality, whereas global *Irf3*^-/-^ mice exhibited similar mortality to WT mice but demonstrated accelerated physiological recovery during the resolution phase, indicative of reduced disease severity rather than improved survival. Deep transcriptomic profiling of lung alveolar macrophages (AM), DC1, and DC2 subsets revealed distinct *irf3* and *irf7* dependent gene programs, with *irf7* prominently driving responses in AM and DC2 populations post-IAV infection. Furthermore, scGPT simulations predicted *irf3*-associated regulation of pathways like IL-17 signaling distinct from *irf7*-biased control over Th17 differentiation and JAK-STAT signaling, suggesting a model where *irf3* mainly drives rapid pathogen sensing and defence, whereas *irf7* regulates sustained inflammation and adaptive immune coordination. Cross-species analyses confirmed conserved and divergent *irf3*/*irf7* activities in human myeloid cells. Our findings provide a detailed framework of *irf3*/*irf7* cell-specific functions, illuminating their nuanced interplay in orchestrating antiviral defence and offering potential targets for immunomodulation. This knowledge may inform the development of targeted antiviral therapeutic strategies and contribute to a more nuanced understanding of innate immune regulation.

## Introduction

Type I interferons (IFN-I) serve as critical mediators of antiviral immunity 1. During influenza A virus (IAV) infection, their proper regulation through interferon regulatory factors (IRFs) is essential for mounting effective immune responses while preventing excessive inflammation 2. Two key members of the IRF family, *irf3* and *irf7*, play central yet distinct roles in this process 3-5. Emerging evidence suggests that these transcription factors may function differently across immune cell populations, although their cell type-specific roles remain incompletely understood 6-8. The initial recognition of viral components occurs through pattern recognition receptors (PRRs), particularly Toll-like receptors (TLRs) 9-11. TLR3 detects double-stranded RNA in endosomes, whereas TLR7 recognizes single-stranded viral RNA 12. These distinct recognition events engage different adaptor molecules: TLR3 signals via TRIF, while TLR7 utilizes MyD88 13-15. Both pathways ultimately activate *irf3* and *irf7*, though their relative importance varies by cell type 4, 16. For example, plasmacytoid DCs (pDCs) rely heavily on the TLR7-MyD88-*irf7* axis 3, while conventional DCs (cDCs) and macrophages employ both TRIF and MyD88-dependent pathways 7.

*Irf3* and *irf7* exhibit distinct regulatory patterns 3, 17. *Irf3* is constitutively expressed and rapidly activated via TBK1-mediated phosphorylation upon viral detection 18, 19. This activation requires STING-dependent pathways in some contexts 20. In contrast, *irf7* typically shows low basal expression but is strongly induced by IFN-I signaling, creating a positive feedback loop 3. Recent studies have revealed that both factors can be activated through multiple pathways, suggesting additional complexity in their regulation 6, 8. Moreover, recent technological advances, particularly single-cell RNA sequencing and spatial transcriptomics, have revealed unprecedented heterogeneity in the immune response to IAV 21-24. These studies have identified distinct subpopulations of macrophages and DCs with unique transcriptional profiles within specific tissue microenvironments, suggesting complex regulation of antiviral immunity 21, 22.

The cell type-specific functions of *irf3* and *irf7* are particularly evident in specialized immune populations 4, 7. In pDCs, *irf7* is essential for robust IFN-I production through MyD88-dependent signaling 3, 25. cDCs show differential requirements for *irf3* and *irf7*, with distinct subsets exhibiting unique activation patterns 5, 7, 18. Macrophages display yet another pattern of *irf3*/7 dependency, with alveolar macrophage (AM) subsets showing unique responses to IAV infection 26. Macrophages and dendritic cells (DCs) also differ fundamentally in their interaction with IAV. While macrophages typically support abortive viral replication 27, DCs support productive infection and may facilitate viral dissemination 28. These differences likely reflect distinct evolutionary pressures and functional specializations 29, however, the molecular mechanisms underlying these differences remain poorly understood.

Several key questions remain regarding the roles of *irf3* and *irf7* in antiviral immunity. While both transcription factors are known to regulate IFN-I production, how macrophages and DCs differentially respond to distinct TLR stimuli in an *irf3*/7-dependent manner remains unclear 2, 30. The precise molecular mechanisms governing the nuclear translocation and activation of *irf3* and *irf7* in macrophages versus DCs have not been fully elucidated 13, 20. Furthermore, although both *irf3* and *irf7* are implicated in antiviral defense, their relative importance for protective immunity during *in vivo* influenza infection remains controversial 31-33.

In this study, we investigated the hypothesis that *irf3* and *irf7* orchestrate distinct, cell type-specific antiviral programs in macrophages and DCs. Our analysis revealed that *irf3* and *irf7* have distinct and context-dependent regulatory functions in macrophages, with *irf7* unexpectedly restraining *irf3*-dependent IFNβ production, while these factors exhibit complementary roles in DC subsets. Paradoxically, while Irf7 deficiency conferred protection against viral lethality, *Irf3* deficiency resulted in accelerated weight recovery despite comparable mortality rates. We show that *irf3* and *irf7* coordinate cell type-specific transcriptional dynamics in a spatiotemporal manner during influenza infection where *irf3* drives immediate antiviral responses while *irf7* sustains inflammatory programs. These findings challenge the conventional view of *irf3* and *irf7* as redundant antiviral factors and establish a new framework for understanding their spatiotemporally controlled, cell type-specific regulatory networks in viral immunity.

## Results

### A Paradoxical Role for *irf3* in Influenza Pathogenesis is Rooted in Cell-Type-Specific TLR Responses

**Figure 1:**
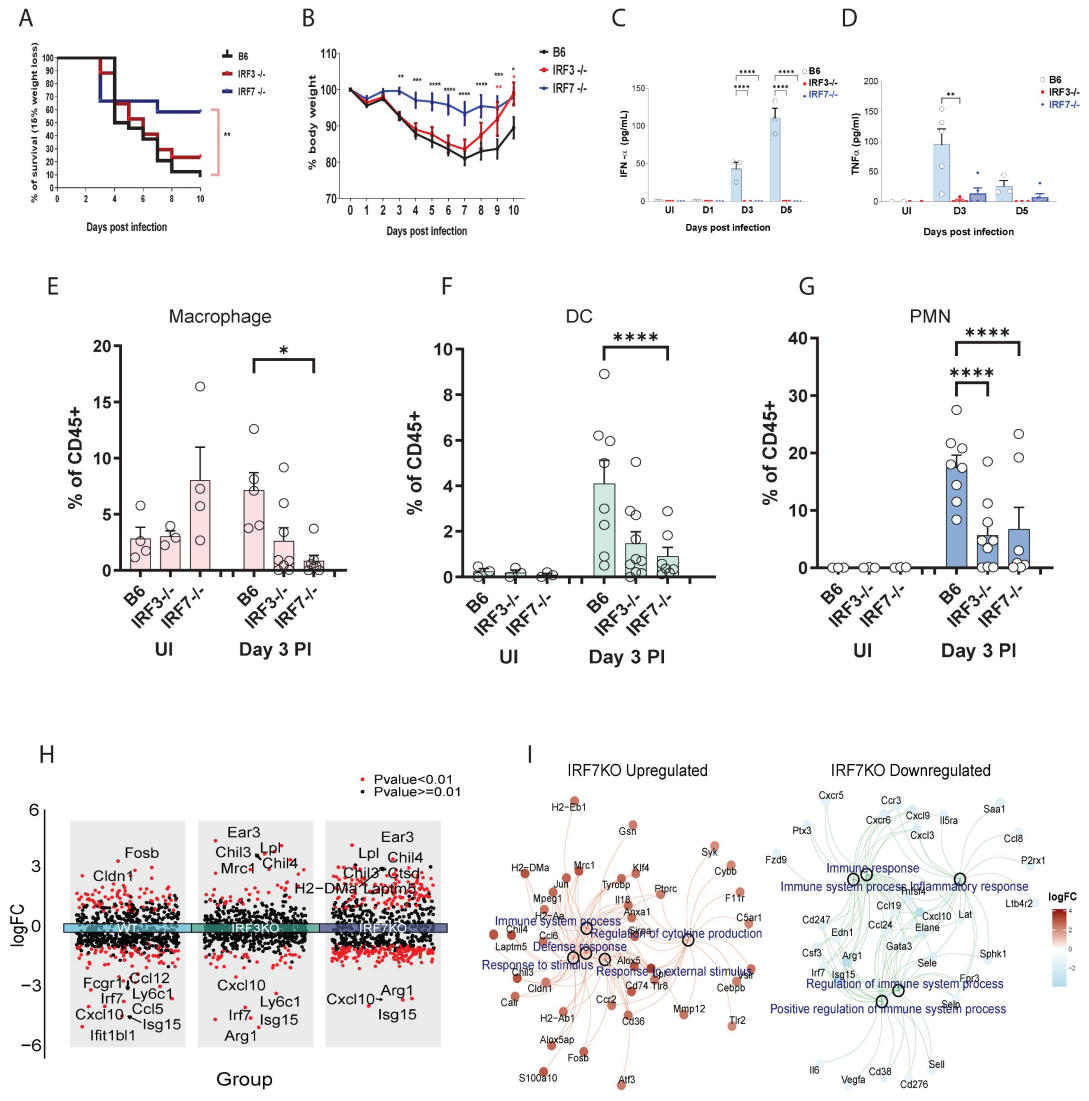
** Distinct outcomes of *irf3* and *irf7* deficiency in influenza pathogenesis and in myeloid cell responses to TLR agonists. (A-G)** *In vivo* assessment of influenza A virus (IAV) pathogenesis in wild-type (B6), *irf3*^⁻/⁻^, and *irf7*^⁻/⁻^ mice. **(A)** Survival curves and **(B)** body weight loss following infection with 500 PFU of A/PR8 IAV (n = 10 mice per group). **(C, D)** IFN-α and TNF-α cytokine levels and **(E-G)** % of leukocytes in bronchoalveolar lavage (BAL) fluid at indicated time points post-infection (n = 5-7 mice per group). **(H)** Volcano plots of differentially expressed genes (influenza infection vs. non-infection) in WT, *irf3*⁻/⁻, and *irf7*⁻/⁻ mice. **(I)** Gene-concept network showing GO biological process enrichment analysis for *irf7* ⁻/⁻ upregulated (left) and downregulated (right) genes. For panels (B-G), significance was determined by one-way ANOVA with Tukey's post-hoc test or Log-rank test: *p < 0.05, **p < 0.01, ****p < 0.0001 vs. wild-type (B6) controls.

We first determined the net contribution of *irf3* and *irf7* transcription factors in virus infection using wild-type (WT), *irf3*^⁻/⁻^, and *irf7*^⁻/⁻^ mice challenged with a lethal dose of influenza A virus (IAV). *irf7*^⁻/⁻^ mice were significantly protected from IAV-induced lethality, whereas *irf3*^⁻/⁻^ mice were similar to WT controls **(Figure 1A)**. A more rapid recovery from virus-induced weight loss was observed in *irf3*^⁻/⁻^ mice but not *irf7*^⁻/⁻^ mice **(Figure 1B)**. This discrepancy was not related to type I interferon or pro-inflammatory cytokine production in the airways, as both *irf7*^⁻/⁻^ and *irf3*^⁻/⁻^ mice exhibited a dampened inflammatory milieu, characterized by significantly lower IFNα and TNFα levels in the bronchoalveolar lavage fluid **(Figure 1C, D)**. Immune cell profiling showed that macrophage proportions were most significantly reduced in *irf7^⁻/⁻^* mice (**Figure 1E**), whereas both DCs and polymorphonuclear neutrophils (PMNs) were decreased in *irf3^⁻/⁻^* mice (**Figure 1F, G**).

To investigate the molecular basis underlying the distinct phenotypes observed between *irf3*^-/-^ and *irf7*^-/-^ mice, we conducted NanoString transcriptional analysis on FACS-sorted lung immune cell subsets, at steady state and following influenza infection. Integrated volcano plots show that distinct genes are upregulated and downregulated at Day 3 post-influenza infection **(Figure 1H** and **Table S1)**. Many more genes are upregulated in the *irf7*⁻/⁻ compared to WT, which could suggest that *irf7* may represses many genes which are normally not observed in the WT mice during infection. GO pathway enrichment analysis of the genes which were regulated by *irf7* revealed that many of the genes were related to defense responses and regulation of immune response **(Figure 1I** and **Table S2)**.

### Transcriptional Profiling Reveals Distinct Functional Signatures of Lung DC Subsets and Alveolar Macrophages at Steady State and during Influenza Infection

**Figure 2:**
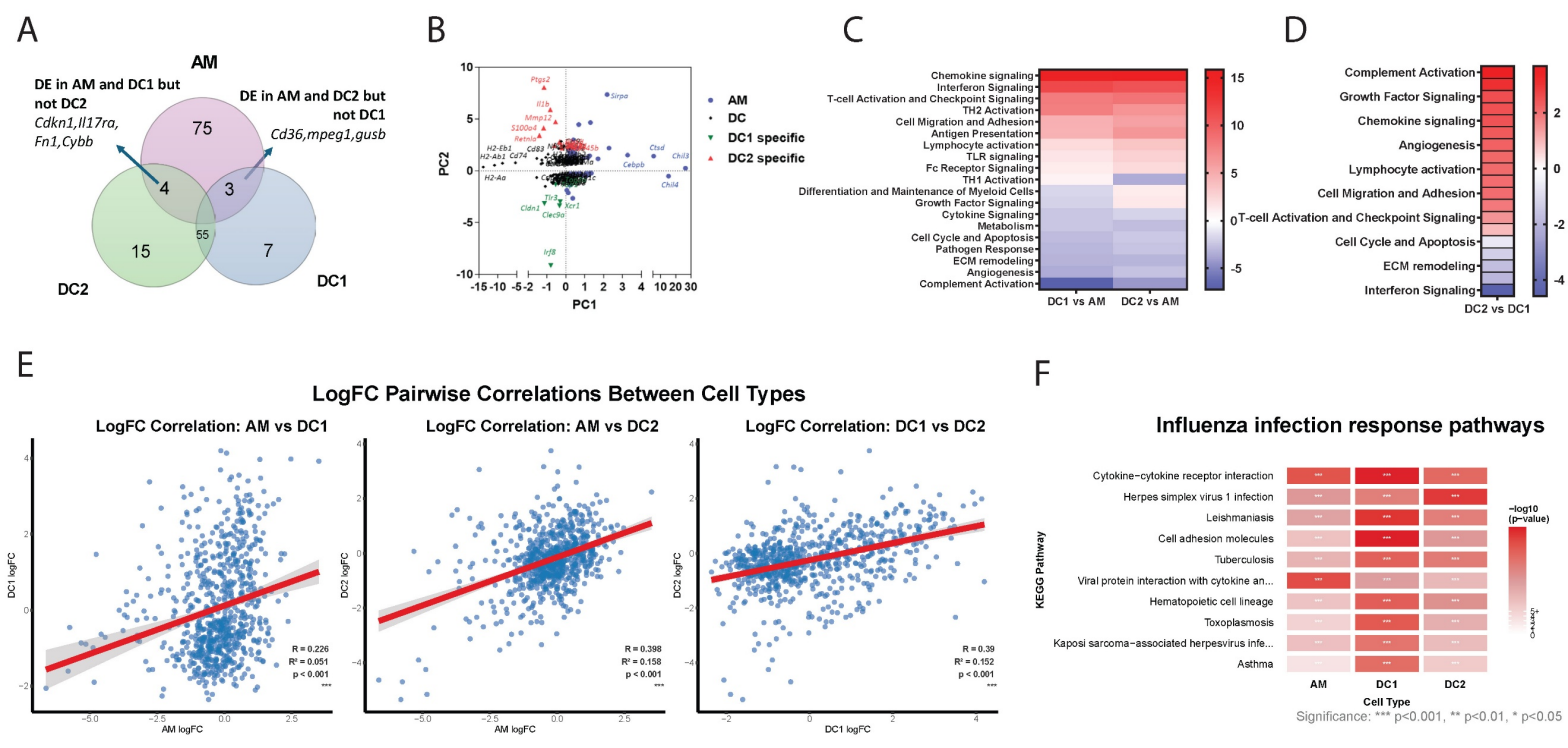
Transcriptional landscape of lung DC subsets and alveolar macrophages at steady state and following influenza infection. **(A)** Venn diagram showing unique and shared gene expression among DC1, DC2, and AM populations at steady state. **(B)** Principal component analysis displaying distinct clustering of AM, DC, DC1-specific, and DC2-specific gene signatures. **(C-D)** Heatmaps comparing directed global significance scores between DC1 vs. AM, DC2 vs. AM, and DC1 vs. DC2. **(E)** Scatter plots showing correlations between log₂ fold changes (logFC) of differentially expressed genes in response to influenza infection. Each point represents a single gene, with logFC values comparing flu-infected vs. uninfected conditions. Linear regression lines (in red) with 95% confidence intervals (gray shading) are shown. Statistical parameters are displayed in each panel: Pearson correlation coefficient (R), coefficient of determination (R²), *p-value*, and significance level (*p < 0.001*). **(F)** Heatmap showing the top 10 immune pathways most significantly enriched across AM, DC1, and DC2 in response to influenza infection. Color intensity represents the strength of antiviral pathway activation (-log₁₀ *p*-value), ranging from white (no activation) to red (strong activation). Statistical significance: * p < 0.05, ** p < 0.01, *** p < 0.001.

We next analyzed the differentially regulated NanoString genes in DC1, DC2, and AM following influenza infection. At steady state, 55 genes were commonly expressed in both DC subtypes while 75 genes were uniquely expressed in AM **(Figure 2A)**. PCA revealed a distinct clustering of AM-specific and DC-specific gene expression profiles **(Figure 2B)**. Within DCs, there are genes commonly expressed by both DC1 and DC2 subtypes, such *as Cd74, Cd83,* and* H2 proteins*. DC1 cells uniquely express genes such as* Irf8* and *Clec9a*, a well-known DC1 marker. In contrast, DC2 cells specifically express *Retnla*, *S100a4*, *IL1b* and *Ptgs2*. Meanwhile, AMs notably express *chil3*/4*, Cebpb* and *Sirpa*. Distinct differences were observed between DCs and macrophages **(Figure 2C)**, particularly in antigen presentation, lymphocyte activation, and chemokine signaling. While complement activation and growth factor signaling, as well as chemokine and growth factor signaling are highly enriched in DC2, interferon signaling predominates in DC1 **(Figure 2D)**. Gene ontology analysis indicated that DCs are also highly enriched in genes related to PI3K activity and chemokine/chemokine receptor activity, while macrophages are more enriched in complement receptor activity and TLR activity **(Figure S1A, B)**. Overall, the distinct expression patterns reflect the specialized functions of each cell type: AMs in tissue repair and immune regulation within the lungs, DC1 cells in antigen presentation and antiviral responses, and DC2 cells in inflammatory responses and immune system activation. These functional differences underscore the diverse roles of AMs, DC1s, and DC2s in maintaining immune homeostasis and responding to pathogens.

Next, we analyzed transcriptional changes in AM, DC1, and DC2 cells isolated 3 days post-influenza infection compared to their steady-state counterparts. NanoString analysis revealed distinct cell-type-specific responses to infection **(Table S3** and **Figure S1C)**. The predominance of upregulated genes suggests DC1 suggests enhanced transcriptional activation consistent with an active antiviral response. The most enhanced genes in DC1 include *Clec9a* and *Cldn1*, and transcription factors *Fos* and *Jun*. Differentially expressed genes in DC2 included chemokines *ccl6* and* ccrl2* and cytokines *Il1r2* and* IL1rl1.* In AM, the most upregulated genes on day 3 post-infection were transcription factors such as *Klf4* and* Egr2,* as well as chemokines such as *Cxcl1***.**

The correlation analysis revealed distinct patterns of transcriptional responses to influenza infection among the three cell types **(Figure 2E)**. AM showed the weakest correlation with DC1 (R = 0.226), indicating that these two cell types exhibit largely independent transcriptional programs in response to influenza infection. In contrast, both AM-DC2 (R = 0.398) and DC1-DC2 (R = 0.390) pairs demonstrated moderate positive correlations, suggesting some shared transcriptional responses between these cell types, with DC subsets showing similar levels of transcriptional coordination. Despite all correlations being statistically significant (*p* < 0.001), the relatively low R² values suggest that each cell type maintains distinct and largely independent transcriptional programs in response to influenza infection.

KEGG pathway enrichment analysis revealed cell type-specific immune responses to influenza infection across AM, DC1, and DC2 **(Table S4** and **Figure 2F)**. Core antiviral immune responses were shared across all three cell types (71 pathways, 58%), highlighting fundamental host defense mechanisms against influenza infection. These included critical antiviral pathways: cytokine-cytokine receptor interactions for immune cell communication, cell adhesion molecules for immune cell trafficking, and viral infection response pathways. Cell type-specific immune specialization was most pronounced in DC1, reflecting its role as a professional antigen-presenting cell.

### *Irf3*/*irf7* Deficiency Analysis Reveals Cell-Type-Specific Transcriptional Dependencies

**Figure 3:**
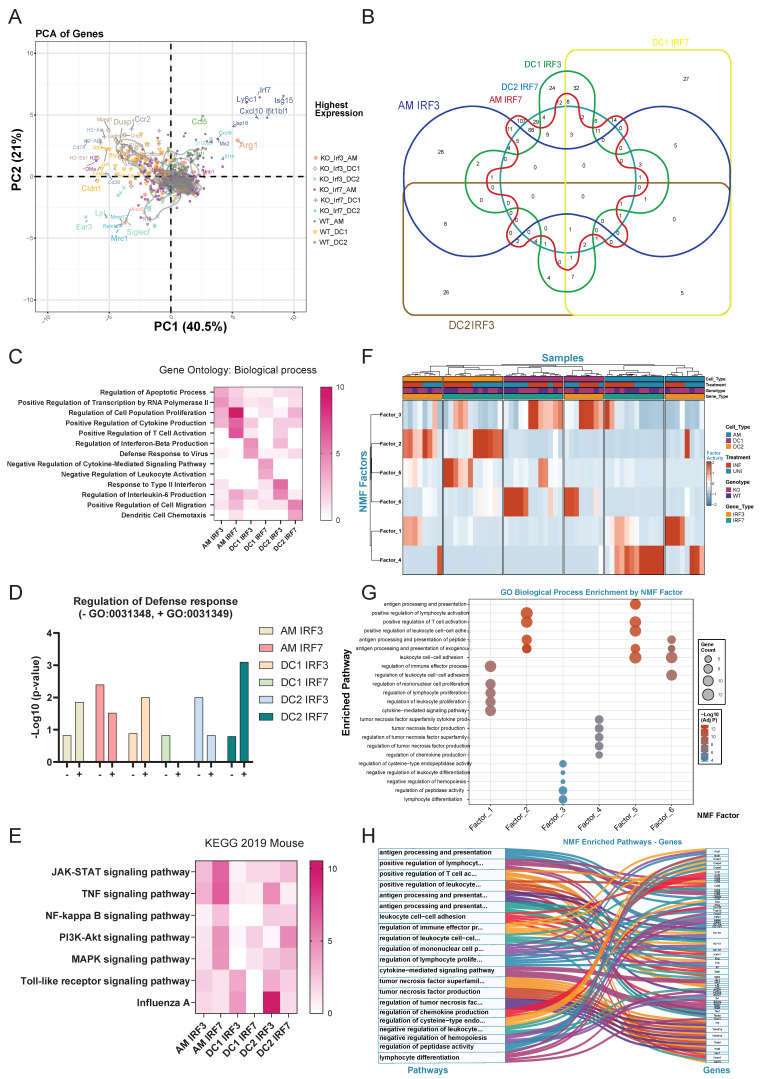
*Irf3* and *irf7* regulate cell type-specific gene expression patterns in alveolar macrophages and DC subsets during influenza infection in mice. **(A)** Principal component analysis (PCA) of gene expression in AM, DC1, and DC2 cells from wild-type, *irf*3^-/-^, and *irf*7^-/-^ mice, showing distinct clustering by cell type and genotype. **(B)** Venn diagram illustrating the overlap of genes requiring *irf3* or *irf7* for expression across AM, DC1, and DC2 populations, with numbers indicating gene counts in each segment. **(C)** Heatmap of GO biological process enrichment for genes dependent on *irf3* or *irf7* in each cell type. Color intensity indicates significance level (-log10 *p*-value). **(D)** Bar graph showing the regulation of defense response in different cell types by *irf3* or *irf7*, with positive and negative regulation of antiviral defense responses. **(E)** Heatmap displaying KEGG pathway enrichment analysis for genes dependent on *irf3* or *irf7* across cell types, highlighting cell type-specific signaling pathway regulation. **(F)** Heatmap showing the normalized activity of six identified NMF factors across lung immune cell subsets (AM, DC1, DC2) from WT, *irf3*^-/-^, and *irf7*^-/-^ mice, under uninfected (UNI) and influenza-infected (INF) conditions. **(G)** Bubble plot showing GO biological process enrichment for top contributing genes of each NMF factor. Dot size indicates gene count; color denotes statistical significance. **(H)** Sankey diagram linking NMF factors to their representative genes and enriched GO biological processes, illustrating the functional organization of NMF-derived transcriptional modules.

PCA of NanoString data from WT, *irf3^-/-^* and *irf7^-/-^* mice revealed distinct clustering patterns based on genotype and cell type **(Figure 3A).** WT samples clustered distinctly from *irf3*^-/-^ and *irf7*^-/-^ counterparts, indicating that loss of either transcription factor significantly alters gene expression landscapes. Key interferon-stimulated genes (*Isg15, Ifit1bl1, Mx2, Cxcl10*) were highly expressed in WT samples, particularly in AM and DC2, demonstrating robust interferon responses that were diminished in both knockout conditions. Cell type-specific signatures were apparent: AM samples associated with *Arg1, Alox5*, and *Siglec-f* expression; DC1 with MHC class II genes (*H2-Aa, H2-Ab1, H2-Eb1*) and CD74; and DC2 with *Mrc1* and *Cldn1*. Notably, inflammatory mediators (*Il1b, Il6, Il18*) exhibited minimal differences across genotypes, suggesting that canonical inflammatory cytokine expressionis less affected by *irf3* or *irf7* deficiency than interferon signaling. Further analysis revealed that *irf7* regulates a broad set of genes in AM and DC2 cells, while *irf3* modulates a narrower set of genes with more specialized functions **(Figure 3B)**.

Gene ontology enrichment analysis identified distinct patterns of gene regulation by *irf3* and *irf7* deficiency in AM, DC1 and DC2 following influenza infection (**Figure 3C**). In AM, *irf3* is linked to transcription and apoptosis while *irf7* regulates proliferation, cytokine production and T cell activation. On the other hand, *irf3* is associated with cytokine production and anti-viral defense responses in DC1 and DC2, while *irf7* is involved in negative regulation of cytokine signaling and leukocyte activation in DC1 as well as DC migration in DC2. As shown in** Figure 3D,** genes in AMs and DC1 dependent on *irf3* are more involved in the positive regulation against virus while those dependent on *irf7* are enriched more in the negative regulation. In contrast, in DC2, *irf3*-dependent genes and *irf7* dependent genes are more involved in negative and positive regulation of defense, respectively. Overall, our NanoString data show that *irf7* generally controls a wider range of genes and pathways, including key signaling pathways like JAK-STAT and PI3K **(Figure 3E)**.

Non-negative Matrix Factorization (NMF) decomposition identified six distinct factors that captured the major axes of transcriptional variance after infection **(Figure 3F and Supplementary Methods).** Factor activity analysis revealed that *irf3* and *irf7* possess distinct cell-type-specific roles dependent on infection status** (Figure 3F and Table S5)**. *irf7* was critical for the activation of adaptive immune programs (Factor 5) in DC2s during influenza infection, while it also enriched a specific inflammatory module (Factor 4) in AM at steady state. In contrast, *irf3* deficiency increased the activity of an immediate inflammatory program (Factor 1) in infected macrophages. Furthermore, loss of either transcription factor induced a compensatory pathway (Factor 3) in DC1s, highlighting their distinct and non-redundant roles in immune regulation.

A network visualization and pathway-gene relationships **(Figure 3G, 3H)** showed that *irf3* and *irf7* coordinate cell-type-specific immune programs and organize distinct functional clusters. Factors 1 and 4 are predominantly active in macrophages for immediate inflammatory responses, while Factors 2, 5, and 6 drive DC functions in antigen presentation and T cell priming during influenza infection. These data show that *irf7* is important in antigen processing and presentation while *irf3* may play more of a role in immediate inflammatory responses.

### Distinct Transcriptional Responses in Macrophages and DCs to TLR Agonists

**Figure 4:**
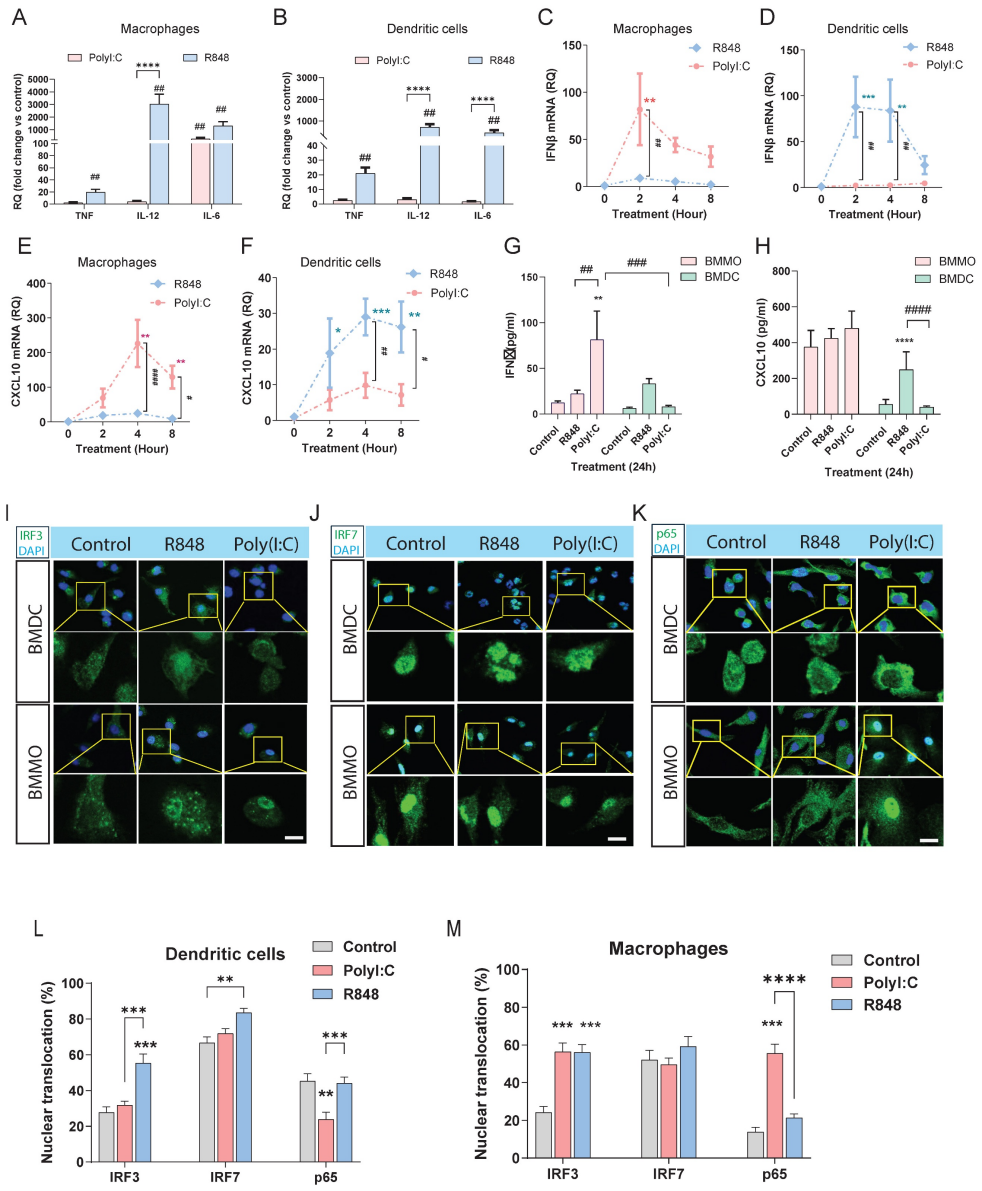
** R848 and Poly(I:C) differentially regulate innate immune responses in bone marrow-derived macrophages and DCs.** Analysis of *in vitro* responses in BMMOs and BMDCs following TLR stimulation. **(A, B)** Real-time qPCR analysis of *Tnf*, *Il-12*, and *Il-6* mRNA after 4-hour stimulation with R848 (TLR7 agonist) or Poly(I:C). **(C, D)** Time-course analysis of *Ifnb1* mRNA and **(E, F)** *Cxcl10* mRNA in BMMOs and BMDCs stimulated as indicated. **(G, H)** IFN-β and CXCL10 protein levels in culture supernatants after 24-hour stimulation. **(I-K)** Representative immunofluorescence images of IRF3 **(I),** IRF7 **(J),** and p65 **(K)** localization in BMDC and BMMO under control conditions or after 1-hour treatment with R848 or Poly(I:C). Scale bars: 10 μm. Insets show magnified views of nuclear regions**. (L-M)** Quantification of nuclear fluorescence intensity for IRF3, IRF7, and p65 in BMMO and BMDC under different treatments. n ≥ 50 cells per condition from three independent experiments. For panels (A-H), significance was determined by two-way ANOVA with Dunnett's multiple comparisons test: # p < 0.05, ## p < 0.01 vs. control; *p < 0.05, **p < 0.01, ****p < 0.0001 vs. compared groups. All data are presented as mean ± SEM.

Our NanoString results above have shown that *irf3* may be involved more in innate anti-viral responses while *irf7* may be more involved in sustained responses such as antigen presentation and T cell activation. In addition, macrophages are thought to be more involved in innate immunity while dendritic cells, as the primary antigen presenting cell, are involved in adaptive immunity. To define the role of *irf3* and *irf7* within macrophages and dendritic cells and to model the interaction with viral dsRNA and ssRNA, we systematically compared the responses of bone marrow-derived macrophages (BMMOs) and DCs (BMDCs) to stimulation with the TLR3 agonist Poly(I:C), alongside the TLR7 agonist R848. While both BMMOs and BMDCs upregulated pro-inflammatory cytokines like *Tnf, Il-12* and *Il-6* in response to R848, this response was significantly weaker upon Poly(I:C) stimulation **(Figure 4A, B)**. When we examined the IFN-I axis, we observed that macrophages were more sensitive to Poly(I:C), launching a potent and rapid *Ifnb1* transcriptional response that was significantly stronger than their response to R848 **(Figure 4C)**. In contrast, BMDCs were largely unresponsive to Poly(I:C), failing to induce significant *Ifnb1* transcription, which was lower than their response to the TLR7 agonist R848 **(Figure 4D)**. This differential IFNβ induction was largely mirrored in the expression of the canonical interferon-stimulated gene (ISG), *Cxcl10*. Macrophage *Cxcl10* expression was robustly induced by Poly(I:C) **(Figure 4E)**, whereas *Cxcl10* was not significantly induced in DCs **(Figure 4F)**. The secretion of IFNβ and CXCL10 at 24 hours confirmed these cell-specific patterns **(Figure 4G, H),** with DCs producing much fewer cytokines and being more sensitive to TLR7 vs TLR3 stimulation. These results support the model that macrophages are more sensitive to TLR3/* irf3* while DCs may be more responsive to TLR7/*irf7*.

To further dissect the transcription factors which could be regulated in BMMO and BMDCs after TLR3 and TLR7 activation, we next examined nuclear translocation of IRF3, IRF7, and p65 after 1h stimulation with Poly(I:C) or R848 by immunofluorescence staining **(Figure 4I-M).** Firstly, IRF7 showed detectable nuclear localization under basal conditions in both BMDC and BMMO. Following R848 stimulation, both IRF3 and IRF7 translocate to the nucleus in DCs **(Figure 4I- L)**, while in macrophages, IRF3, but not IRF7 translocates to the nucleus **(Figure 4I, J, M)**. However, after Poly(I:C) stimulation, neither IRF3 nor IRF7 translocated; surprisingly, p65 translocation was inhibited, which could explain the suboptimal activation of DCs by Poly(I:C) **(Figure 4I, J, K, L)**. In macrophages, we observed that only IRF3 translocates to the nucleus after Poly(I:C) stimulation with no translocation of IRF7, whereas both IRF3 and p65, but not IRF7 translocates to the nucleus after R848 stimulation **(Figure 4I, J, K, M)**. These findings demonstrate stimulus-dependent nuclear translocation of key transcription factors which is cell-type specific, with R848 predominantly promoting IRF3 and IRF7 nuclear localization in DCs, but not macrophages, and Poly(I:C) inhibiting p65 in DCs, while activating p65 in macrophages. Collectively, these data establish that macrophages and DCs possess fundamentally distinct intrinsic programs in response to TLR7 and TLR3 stimulation.

### Distinct IRF Requirements for Anti-Viral and Pro-Inflammatory Cytokine Production in Macrophages versus DCs

**Figure 5:**
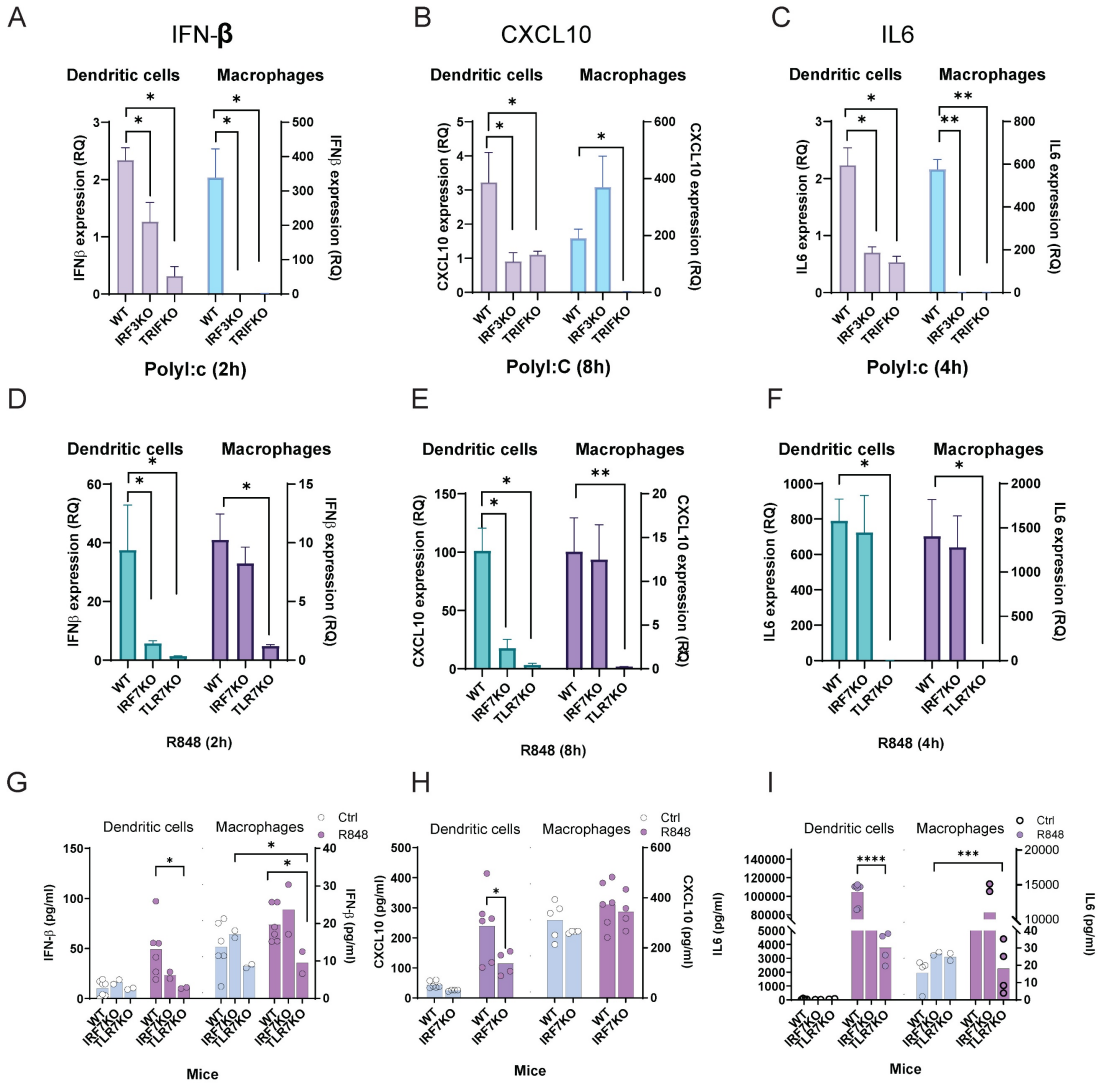
** Roles of *irf3* and *irf7* in bone marrow-derived cells in response to Poly(I:C) and R848 stimulation. (A-C)** IFNβ, CXCL10 and IL6 expression in WT, irf3 ^-/-^ and trif ^-/-^ dendritic cells or macrophages after Poly(I:C) stimulation at the indicated times. **(D-F)** ifnβ, cxcl10 and il6 expression in WT, irf7^-/-^ and tlr7^-/-^ dendritic cells or macrophages after R848 stimulation at the indicated times. **(G-I)** IFNβ, CXCL10 and IL6 production in WT, irf7 ^-/-^ and tlr7 ^-/-^ dendritic cells or macrophages after R848 stimulation after 24h. Data are presented as mean ± SEM. *p < 0.05, **p < 0.01, **** p< 0.0001 vs. compared groups.

TLR3 predominantly activates *irf3*-dependent pathways, whereas TLR7 stimulation drives *irf7*-mediated cytokine production in both macrophages and DCs. We next explored the contribution of these known signaling pathways after stimulation by TLR3 and TLR7 using WT C57BL6, *irf3* and *irf7* deficient bone marrow derived macrophages or DCs, respectively. In BMDC, we observed that following a 2-h stimulation with Poly(I:C), IFNβ mRNA expression was increased by ~ 2-fold, which was dependent on *irf3* and TRIF **(Figure 5A)**. This IRF3- and TRIF-dependent induction of Ifnb1 was also reflected in subsequent expression of Cxcl10 at 8 h and Il6 at 4 h **(Figure 5B, 5C)**. In BMMO, a similar dependence on *irf3* and TRIF was observed after TLR3 stimulation for IFNβ **(Figure 5A)** and *il6* **(Figure 5C)**. However, CXCL10 expression was not dependent on *irf3*, as *irf3 ⁻/⁻* BMMO exhibited a similar expression of CXCL10 after Poly(I:C) treatment **(Figure 5B)**, suggesting other transcription factors may be more important in ISG production downstream of type-1 interferons. After R848 stimulation, BMDC exhibited dependence on *irf7* and TLR7 for IFNβ and *cxcl10* expression **(Figure 5D, E)**, but not *il6* expression **(Figure 5F)**. In contrast, R848 stimulation in BMMO was not dependent on *irf7* for *ifnβ, cxcl10* or *il6* expression **(Figure 5D-F)**. This observation for R848 was translated into cytokine production for IFNβ, CXCL10 and IL-6, where *irf7* is at least partially important for IFNβ and CXCL10 production, but not IL-6 in BMDC, while in BMMO, *irf7* is not required for cytokine production after TLR7 activation **(Figure 5G-I)**. Together, these findings highlight distinct IRF-dependent signaling mechanisms upon TLR3 and TLR7 stimulation which are different in macrophages and DCs.

### Integrated Dataset Analysis Validates IRF Signaling Patterns across Experimental Platforms during Influenza Infection

**Figure 6:**
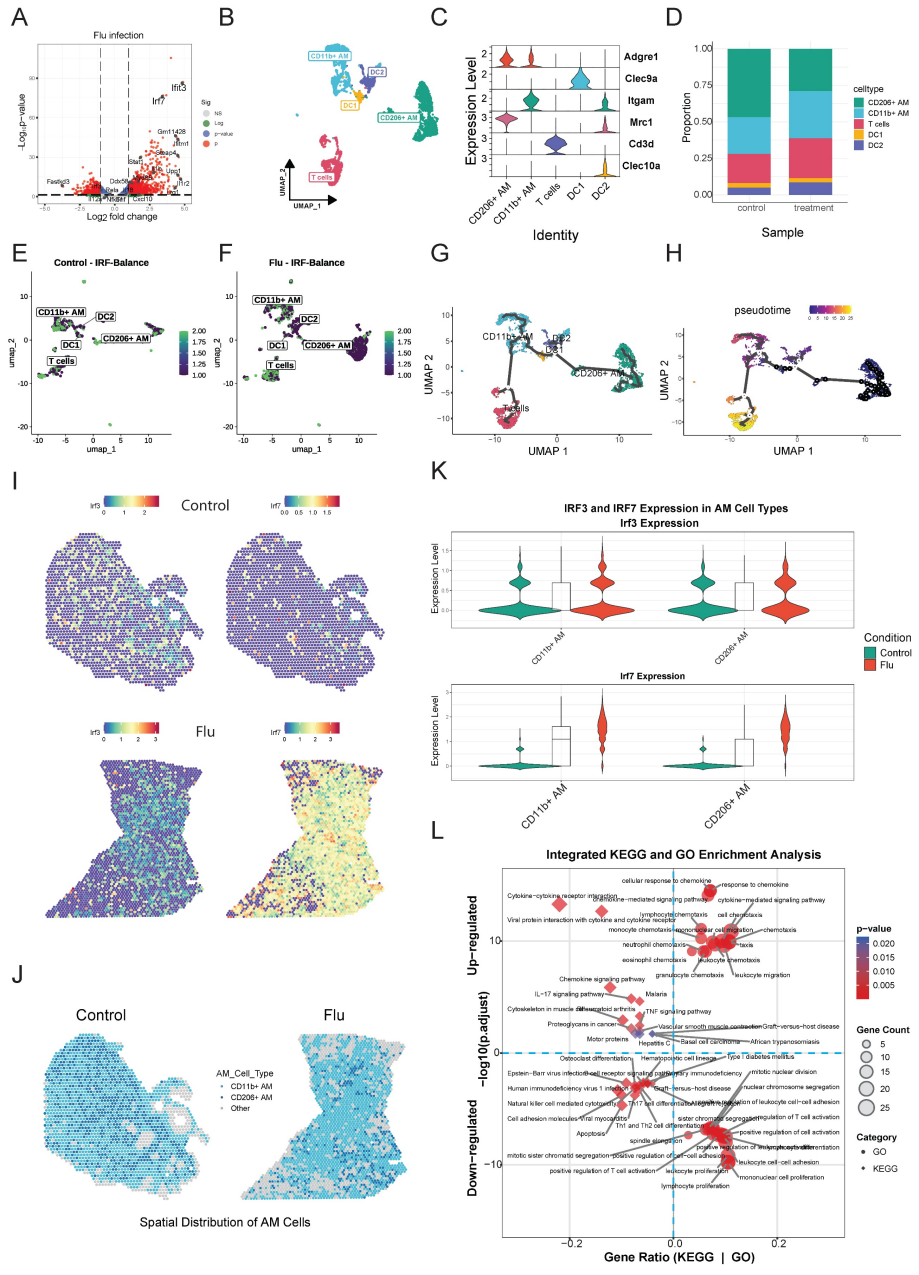
** Integrated analysis reveals the different patterns of *irf3* and *irf7* activity in macrophages and dendritic cells during influenza infection. (A)** Volcano plot showing differential gene expression in influenza-infected mice compared to controls in Bulk RNA analysis. Genes with significant changes (adjusted *p-value* < 0.05 and |log2FC | > 1) are highlighted, with key genes such as Irf3, Irf7, Il12a, Cxcl10, Stat1, and Il1b annotated.** (B-D)** Single-cell transcriptomics analysis of influenza-infected mouse lung tissue.** (B)** UMAP visualization of cell clusters. **(C)** Violin plots depicting expression levels of marker genes across cell types. **(D)** Bar plot showing changes in cell type proportions between control and influenza-infected samples. **(E,F)** UMAP projection of the IRF-Balance Index in control (left) and influenza-infected (right) conditions. **(G,H)** Trajectory analysis of single-cell RNA-seq data showing inferred lineage relationships. **(I)** Visualization of *irf3* and* irf7* in spatial transcriptomics data from control and influenza-infected lung samples. **(J)** Spatial distribution of major AMs cell types in control and influenza-infected lung sections, highlighting the infiltration of immune cells. **(K)** Violin plots showing the expression of *Irf3* and *Irf7* in CD11b+ and CD206+ AMs under control and influenza conditions. **(L)** Integrated KEGG and GO pathway enrichment analysis of differentially expressed genes in AMs. The x-axis represents the gene ratio, and the y-axis shows the -log10(adjusted p-value). Point size indicates gene count, and color represents the p-value.

To validate our results, we analyzed transcriptomic data from influenza-infected mouse lung samples (GSE124404). Differential gene expression analysis showed significant upregulation of anti-viral and inflammatory genes, including *irf3, irf7, Il12a, Cxcl10, Stat1*, and *Il1b* **(Figure 6A** and **Table S6)**. KEGG pathway analysis highlighted activation of immune response pathways, including IFN-α/β signaling, cytokine signaling, and pathogen recognition receptor signaling **(Figure S2A** and **Table S7)**. However, pathways involved in T cell differentiation and function, such as Th1, Th2 and Th17 cell differentiation, were downregulated.

UMAP analysis of single-cell transcriptomics data from lungs of influenza-infected mice (**GSE228594**) identified distinct cell type clusters **(Figure S2B, C)**. Following infection, a slight increase in the proportions of DC1 and DC2 cells was observed **(Figure S2D)**. Focusing on macrophages and DC, including CD11b+ AM (M1) or CD206+ AM (M2) **(Figure 6B, C)**, the data showed that infection increased the proportion of M1, T cell and DC2 cells and decreased M2 **(Figure 6D).** Pro-inflammatory cytokines (*Tnf, Il1b, Il6, Il12a, Il18*) were mainly expressed at higher levels in AM **(Figure S2E)**.

Mapping the IRF-Balance Index** (Supplementary Methods)** onto UMAP showed a shift toward *irf7*-dominant activity following influenza infection, particularly in M1 and DC2 cells **(Figure 6E, F;** and **Table S8)**. In contrast, M2 and T cells maintained relatively balanced *irf3*/*irf7* activity.

Trajectory and pseudotime mapping indicated that DC2 cells may act as precursors to both DC1 and AM lineages** (Figure 6G, H;** and **Figure S2F).** *Ifnb1* expression peaked early, particularly in DC2 cells and CD11b+AM, while ISGs like *Cxcl10* showed sustained expression in M1. Cytokine gene expression varied, with *Il12a* peaking early in DCs, while *Il6* and *Tnf* expression was maintained across cell types. *irf3* expression remained stable, whereas *irf7* expression gradually increased in M1 cells over time. DC1 and DC2 shared similar but quantitatively distinct profiles, while M1 and M2 displayed unique expression patterns. Notably, IFN- I and CXCL10 expression were primarily induced in M1.

To further examine *irf3* and *irf7* expression patterns during influenza infection, we integrated mouse single-cell and spatial transcriptomics datasets from influenza-infected mouse lungs (GSE202322, GSE228594). Due to insufficient dendritic cell numbers in the spatial data, our analysis is focused on AM. Spatial mapping revealed that *irf7* was strongly upregulated across distinct lung regions whereas *irf3* expression was more uniformly distributed **(Figure 6I)**. Analysis of AM subpopulations showed a shift towards more M1 and less M2 after infection suggesting macrophage polarization to a more inflammatory phenotype **(Figure 6J)**. Violin plots demonstrated significantly higher *irf7* expression in M1, while *irf3* expression was moderately elevated in both AM subtypes after infection **(Figure 6K)**. Upregulated genes in AM included those involved in cell cycle regulation (*Ccna2, Mki67, Cdca3*) and innate immunity (*Sh2d1b1, Hmmr, Sept6*), indicating enhanced immune cell proliferation and cytotoxic activity **(Figure S2G).**

Spatial transcriptomics at day 9 post-influenza infection demonstrated *irf3* and *irf7* occupied distinct tissue niches. *irf3* expression was widespread and diffuse while *irf7* was restricted to localized regions of intense activity **(Figure S3A, B)**. Neither factor correlated with high-infection zones, and spatial analysis indicated no significant clustering **(Figure S3C)**, indicating that their activation depends on cell-intrinsic programs within specific microenvironments during infection. Integrated enrichment analysis of KEGG and GO pathways highlighted significant activation of cytokine-cytokine receptor interaction, chemokine signaling, and IFN- I responses in AMs **(Figure 6L)**.

### AI-driven modeling predicts IRF-biased regulatory networks in viral immunity

To overcome the limitations of traditional gene expression analysis, such as limited gene coverage and dropout noise, we used scGPT to simulate transcriptomic responses of individual immune cell types (AM, DC1, DC2) in WT, *irf3*^-/-^ and *irf7*^-/-^ mice after influenza infection. This approach enabled us to identify previously undetectable ISGs, evaluate the impact of genetic perturbations on cellular responses, and map the divergent roles of *irf3* and *irf7* at single-cell resolution.

**Figure 7:**
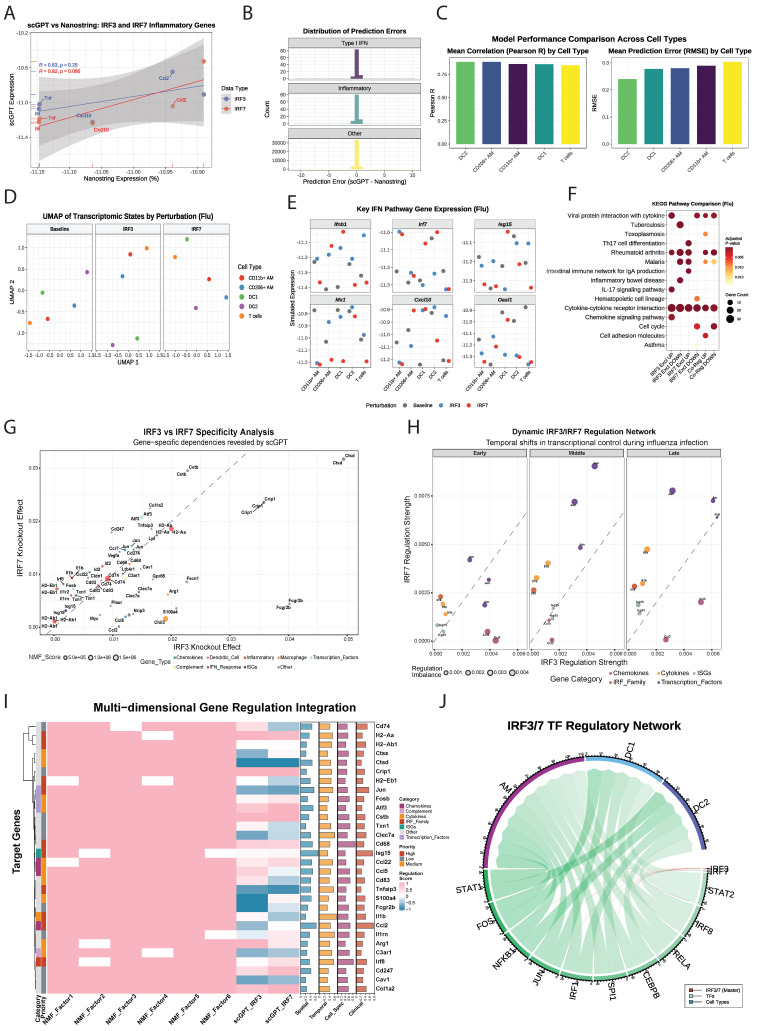
** The scGPT model predicted cell type-specific transcriptomic responses to simulated *irf3*/*irf7* knockout during influenza infection. (A)** Representative scatter plot comparing scGPT-predicted expression versus NanoString measured expression for common genes in CD11b+ AM simulating *irf3* and *irf7* knockout under influenza infection conditions. The line indicates linear regression fit, and the Pearson correlation coefficient (R) is shown. **(B)** Histograms showing the distribution of prediction errors (scGPT - NanoString expression) for genes grouped by functional categories (Type I IFN, Inflammatory, TLR Signaling, Other). **(C)** Comparison of scGPT model performance across cell types. Left panel shows the mean Pearson correlation coefficient (R) averaged across conditions and perturbations for each cell type. Right panel shows the corresponding mean Root Mean Square Error (RMSE). **(D)** UMAP visualization of simulated transcriptomic states for five lung immune cell types (CD11b+ AM, CD206+ AM, DC1, DC2, T cells) during simulated influenza infection. **(E)** Point plots showing simulated expression levels of eight key interferon (IFN) pathway genes across the five cell types and three simulated conditions (Baseline, *irf3* ⁻/⁻, *irf7* ⁻/⁻) during influenza infection. Y-axes represent simulated expression on potentially normalized or log-like scale (free scale per gene). **(F)** Comparative KEGG pathway enrichment analysis. Dot plot shows enriched pathways for genes identified as exclusively upregulated/downregulated by *irf3* ("*irf3* Excl UP/DOWN"), exclusively by *irf7* ("*irf7* Excl UP/DOWN"), or co-regulated by both ("Co-Reg UP/DOWN") during simulated influenza infection (aggregated across cell types). Top 15 enriched pathways are shown for each category. **(G)** Scatter plot of scGPT-predicted *irf3* and *irf7* knockout effects across 72 genes in immune cells during influenza infection. Each point represents a single gene, with *irf3* regulation strength (x-axis) and *irf7* regulation strength (y-axis). Diagonal dashed line indicates equal *irf3*/*irf7* regulation. **(H)** Three-panel temporal analysis showing *irf3* and *irf7* regulation strength during early (2-6h), middle (12-24h), and late (48-72h) phases of influenza infection. Each point represents a gene positioned by *irf3* (x-axis) and *irf7* (y-axis) regulation strength. Point size indicates regulation imbalance magnitude. **(I)** Integration of 30 genes across eight analytical dimensions: six NMF factors (Factor 1-6), scGPT *irf3*/*irf7* predictions, and four validation metrics. Left annotations show gene functional categories and clinical priority levels. Right bar plots display validation dimensions: Spatial expression (blue). Heatmap colors represent standardized regulation scores (blue = negative, white = neutral, red = positive). **(J)** scGPT-Predicted and NMF-Validated *irf3*- and *irf7*-dependent regulatory networks in AM and DC subsets during influenza infection.

We validated the accuracy of the scGPT model by comparing its *in silico* predictions with experimental NanoString data shown earlier. The model's predictions correlated closely with experimental results in representative simulations **(Figure 7A)**, with no systematic bias detected across key functional gene categories **(Figure 7B)**. The predictions were also consistent across all evaluated cell types **(Figure 7C)**. Detailed descriptions of the scGPT framework, fine-tuning process, and additional performance analysis are provided in the **Supplementary Methods and Figure S4**. Collectively, this robust validation confirms that the scGPT model reliably simulates the transcriptomic consequences of genetic perturbations *in silico*.

Simulated knockout of *irf3* or *irf7* resulted in noticeable shifts in the UMAP positions of specific cell types compared to baseline **(Figure 7D).** Analysis of IFN pathway genes showed that simulated expression of canonical ISGs, including *Isg15*, *Mx1*, and *Cxcl10*, was substantially reduced in AM and DCs following deletion of either *irf3* or *irf7* **(Figure 7E).** KEGG pathway enrichment analysis of genes regulated exclusively by *irf3* or *irf7*, or both during influenza infection revealed distinct regulatory roles **(Figure 7F).** *irf3*-exclusive targets were enriched in the IL-17 pathway while *irf7*-specific targets were associated with Th17 cell differentiation, JAK-STAT signaling, and PI3K signaling pathways, in line with our NanoString data. These findings suggest *irf3* mainly supports rapid antiviral defense and pathogen sensing, whereas *irf7* primarily regulates cytokine signaling and adaptive immune coordination.

### Multi-dimensional Integration Reveals Dynamic *irf3*/*irf7* Regulatory Dependencies

The integration of scGPT knockout simulations with NMF analysis provided a detailed view of gene-specific regulatory patterns **(**Figure 7G and **Supplementary Methods)**. Macrophage-associated genes, particularly *Arg1* and *Cd68*, clustered distinctly from other gene categories in the regulatory landscape, suggesting specialized regulatory programs within myeloid cell populations. These genes were mainly associated with NMF Factor 1 and Factor 4, which were most active in AM during influenza infection. The analysis showed that gene regulation was more complex than simple transcriptional redundancy as 44.4% of genes were predominantly regulated by *irf3*, 55.6% by *irf7* and genes with balanced *irf3/irf7* regulation (near the diagonal) were enriched in Factor 2 and Factor 5, which were predominantly active in DCs.

Time-resolved analysis revealed a temporal shift from *irf3 to irf7* dominance during influenza infection **(Figure 7H)**. In the early phase (2-6 hours), *irf3* was central, promoting key chemokines like *Ccl2* and *Ccl5* to initiate immune cell recruitment. In the middle (12-24 hours) and late (48-72 hours) phases, *irf7* became more dominant, sustaining inflammatory responses and expression of ISGs, such as *Il1b* and *Isg15*. Again, this analysis demonstrates that *irf3* primarily initiates the acute response, while *irf7* drives the subsequent inflammatory amplification.

A multi-dimensional framework was developed by integrating NMF analysis, scGPT predictions, spatial-temporal patterns, and clinical data **(Figure 7I, Table S9)**. Six regulatory factors were identified, with Factor 2 and Factor 6 accounting for the majority of variance. Factor 6 was enriched for IRF genes involved in antigen presentation (*Cd74*, *H2-Aa*, *H2-Ab1*), while Factor 2 contained MHC class II components, suggesting coordinated regulation of adaptive immunity. Spatial analysis showed that 90% of genes had cell type-restricted expression, reflecting highly specialized immune compartmentalization. Temporal analysis identified early-response genes (score ≤0.4) and sustained-expression genes (0.4-0.7), but no late-phase genes. Clinical integration showed that genes like *Ccl2* (score: 0.781) were broadly expressed and highly cell-specific (0.663), whereas others like *Il1b* showed restricted expression despite high temporal dynamics (0.672). The multi-dimensional analysis **(Figure 7I)** uncovered three functionally specialized modules: an *irf3*-dominant module responsible for rapid chemotactic responses, an *irf7*-dominant module sustaining inflammation and a co-regulated module maintaining immune balance. These results illustrate that *irf3* and *irf7* organize the immune response into complementary and spatiotemporally controlled programs, where *irf3* initiates rapid recruitment and surveillance, whereas *irf7* ensures ongoing inflammation and adaptive immune engagement.

To further clarify the molecular mechanisms through which *irf3* and *irf7* regulate immune responses in AM and DCs during influenza infection, we constructed a chord diagram modeling the hierarchical network (**Figure 7J**). This model shows that *irf3* and *irf7* act as master regulators initiating distinct and divergent signaling cascades. Edge-weighted interaction analysis indicated that *irf7* predominantly regulates STAT2 in AM and DC1, consistent with its established involvement in JAK-STAT signaling. Conversely, *irf3* regulates a separate group of downstream transcription factors, including IRF8, RELA, and CEBPB. These transcription factors mediate cell-specific effects, with IRF8 mainly influencing AM and DC2, and functions downstream of *irf3* signaling.

NMF analysis supported this hierarchical structure, showing that *irf3* targets (e.g., *Ccl2, Jun*) are enriched in Factor 1, linked to proliferation and inflammatory signaling in AM, while Factor 4 (macrophage-associated response) highlighted *irf7*'s role in restraint TNF signaling. Overall, these findings describe a complex, cell-type-specific regulatory system where *irf3* triggers immediate antiviral responses, while *irf7* modulates longer-term inflammatory and adaptive programs. This framework helps to explain the functional diversity observed among myeloid cells during antiviral immunity.

### Comparative analysis reveals evolutionary conservation of IRF functions across species and viruses

**Figure 8:**
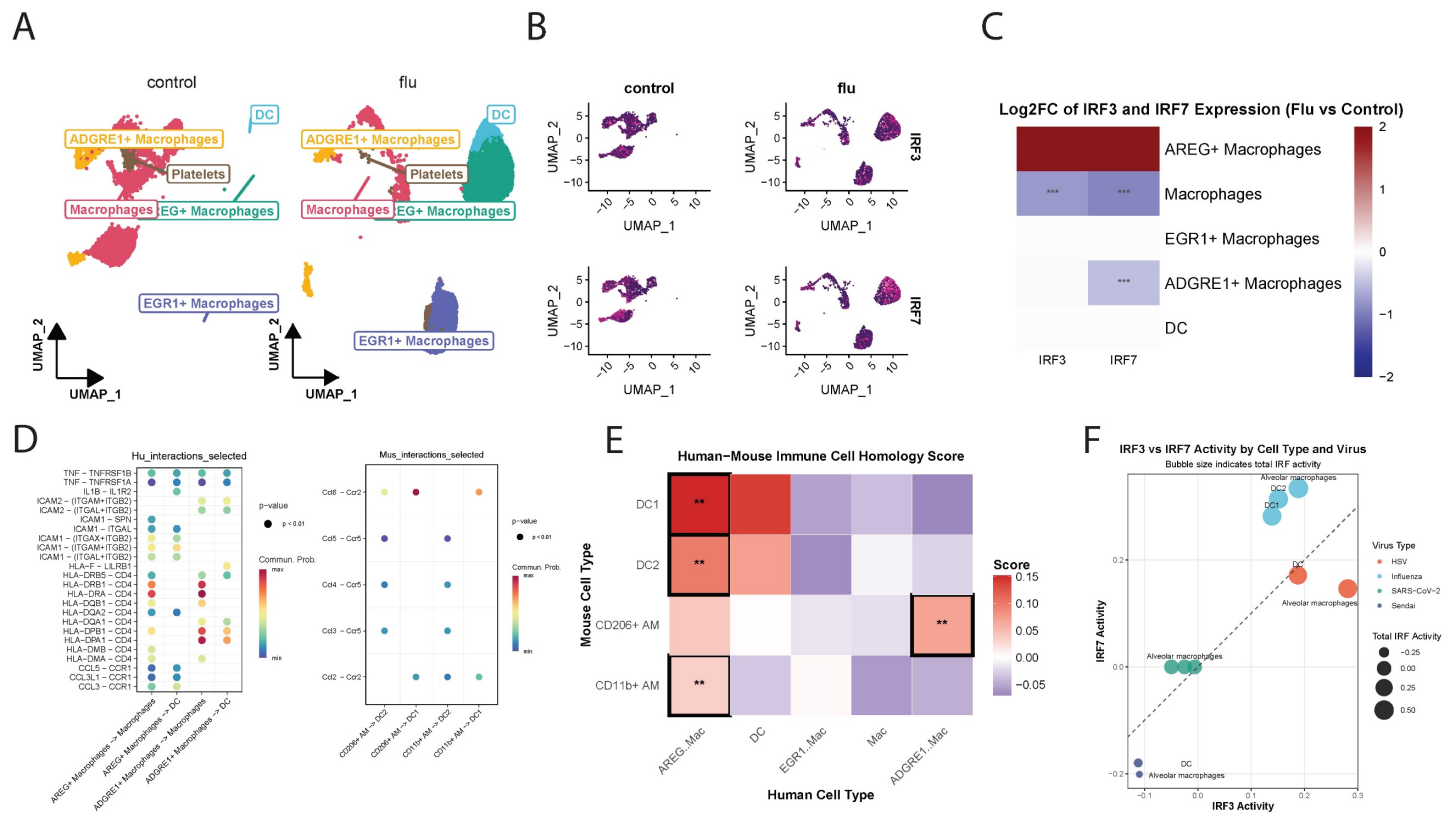
** Comparative analysis of *irf3* and *irf7* in human and mouse macrophages and DCs under influenza infection. (A)** UMAP visualization of unsupervised clustering showing the distribution of immune cell populations in control (left) and influenza-infected (right) human lung samples. **(B)** Feature plots showing UMAP projections of single-cell RNA expression levels of *irf3* and *irf7* in control (left) and influenza-infected (right) lung samples. **(C)** Heatmap showing *irf3* and *irf7* expression differences across cell types and conditions (influenza vs control). **(D)** Dot plots representing ligand-receptor interaction analysis between macrophages and DCs in human (left) and mouse (right) samples using intercellular communication analysis. **(E)** SAMap analysis resulting in mapping scores that quantify the transcriptional similarity between cell populations across the two species. **(F)** *irf3* and *irf7* activity in response to different viral infections. Bubble size indicates the total combined IRF activity (*irf3* + *irf7*), and colors represent different viral infections. The dashed diagonal line indicates equal *irf3* and *irf7* activation. Cell types positioned above the line show *irf7*-dominant responses, while those below exhibit *irf3*-dominant responses. Values represent mean pathway scores from gene expression analysis.

To extend our findings to humans, we analyzed single cell RNA-sequencing data from control and influenza-infected human lung samples (GSE149689). UMAP analysis identified distinct clusters of macrophage subpopulations and DCs, though DCs were present in smaller proportions. In control lungs, there were mostly non-polarized M0 macrophages and AM, whereas influenza-infected lungs showed an expansion of AREG^+^M1 macrophages (M1) and EGR1^+^M2-like macrophages (M2) and DCs (Figure 8A and Figure S5A). High *irf3* expression was observed in M2 while both *irf3* and *irf7* were highly expressed in M1 (Figure 8B, C). Pathways related to cytokine-cytokine receptor interaction, TNF, NF-κB signaling, and IL-17 signaling were significantly enriched in both humans and mice (Figure S5B). However, species-specific differences were noted, with human samples enriched for PD-L1 and PD-1 checkpoint pathways, whereas mouse samples exhibited stronger enrichment in JAK-STAT, Th17 cell differentiation and the NOD-like receptor signaling pathways.

Cell-cell communication analysis demonstrated robust interactions among macrophages and DCs in human lungs, highlighting their prominent role in orchestrating immune responses to infection **(Figure S5C)**. In mice, communication between CD11b^+^ macrophages, DC1, and DC2 was also prominent, though interaction strengths differed compared to humans**.** Ligand-receptor interaction analysis results indicated that human macrophage-DC interactions were significantly enriched for TNF-TNFRSF1, ICAM1-ITGAL, and HLA-related signaling, emphasizing their role in antigen presentation and T cell activation **(Figure 8D).**

SAMap analysis (**Supplementary Methods**) revealed strong homology between mouse and human DC subsets 34 (**Figure 8E**). Mouse DC1 and DC2 populations corresponded closely with human DCs, and also mapped to human AREG+M1 macrophages, suggesting shared activation states during infection. Mouse CD206^+^M2 AMs, (analogous to resident AMs) exhibited the highest similarity with human ADGRE1+ macrophages, supporting their functional equivalence. This alignment enables cross-species comparisons of *irf3*/*irf7* regulated pathways in various myeloid cell populations.

Gene Set Variation Analysis (GSVA) showed virus-specific activation signatures for *irf3* and *irf7* target genes. The balance between *irf3* and *irf7* pathway activation varied substantially between RNA viruses (influenza, SARS-CoV-2, Sendai) and the DNA virus (HSV) **(Figure 8F).** HSV infection induced strong *irf3*-driven responses in DCs and AMs, while RNA viruses induced a more balanced activation of both *irf3* and *irf7* with cell type-specific patterns. AMs showed the highest *irf7* activation during influenza infection, consistent with our earlier findings. Radar plots illustrated that HSV and influenza induce distinct immune activation profiles compared to Sendai virus, with varying engagement of *irf3* and *irf7* pathways. Strong type I IFN responses were observed in AM during HSV and Influenza infection but not Sendai virus, while DCs displayed balanced responses to all viruses **(Figure S5**D**)**. Key antiviral genes including *Isg15*, *Mx1*, and *Rsad2* were most highly expressed during HSV infection, particularly in CD204+ macrophages **(Figure S5E).** Notably, *irf7* expression peaked following HSV infection, whereas *irf3* levels remained constant across all conditions. These findings demonstrate that *irf3*/*irf7* activation is virus and cell-type specific and that the regulatory balance between these transcription factors is a critical determinant of the antiviral immune response to specific pathogens.

## Discussion

*In vivo* influenza infection model revealed unexpected and divergent roles for *irf3* and *irf7* in controlling viral pathogenesis. Notably, mice lacking IRF3 exhibited accelerated recovery from influenza-associated weight loss, rather than enhanced protection from lethality, indicating a reduction in disease severity without a corresponding improvement in survival outcomes 35-37. This finding is particularly intriguing given that previous research has consistently demonstrated the critical role of *irf3* and *irf7* in antiviral responses 3, 38. Previous studies have shown that these transcription factors are essential for the induction of type I interferons and interferon-stimulated genes during viral infection. Thus, the improved clinical recovery observed in *Irf3*-deficient mice suggests that loss of IRF3 does not simply impair antiviral defence but instead reshapes host responses in a manner that limits pathological inflammation. Such effects may involve compensatory activation of alternative antiviral pathways, including contributions from other IRF family members or NF-κB-dependent mechanisms 39, as well as a global attenuation of inflammatory cytokine production that mitigates immunopathology during influenza infection 5, 40.

The observation that *Irf3*-deficient mice recover more rapidly despite overall lethality comparable to wild-type controls highlights a paradoxical, double-edged role for IRF3 in influenza pathogenesis. While IRF3 is classically defined as an essential initiator of antiviral type I interferon responses, it also functions as a potent driver of pro-inflammatory cytokine production. Consistent with this dual role, our data show that *Irf3* deficiency results in a markedly attenuated inflammatory milieu in the airways, including reduced levels of TNF-α and other inflammatory mediators, suggesting that the accelerated recovery observed in surviving *Irf3^-/-^* mice is driven primarily by reduced immunopathology rather than enhanced viral clearance. Importantly, the absence of increased mortality in *Irf3^-/-^* mice indicates that antiviral control remains sufficient to prevent fatal disease. In this context, our integrated analyses identify IRF7, rather than IRF3, as the dominant regulator of protective antiviral programs in alveolar macrophages and DC2 subsets, particularly during later phases of infection. We therefore propose that IRF7-dependent pathways compensate for the loss of IRF3 to maintain viral control below a lethal threshold, whereas IRF3 contributes disproportionately to collateral inflammatory damage that delays recovery. Viewed through this framework, IRF3 deficiency confers a state of enhanced disease tolerance, characterized by reduced tissue damage and faster recovery, without conferring resistance to viral lethality. This distinction reconciles the apparent paradox observed in our *in vivo* data and underscores the divergent roles of IRF3 and IRF7 in balancing antiviral immunity and immunopathology during influenza infection. The differential impact of *irf3* and *irf7* deficiency on immune cell infiltration and cytokine production highlights that, although they have overlapping functions, each may play unique roles in orchestrating the immune response. The hierarchical dependence of lymphocyte infiltration on these factors, with *irf7* playing a more dominant role, adds to our understanding of how these transcription factors shape both innate and adaptive immune responses during viral infection 5, 16, 36. Further examination of their roles in time using scGPT and NMF revealed a clear progression: in the early phase of the immune response, gene regulation is balanced between *irf3* and *irf7* (5 genes each being dominant). In the middle phase, *irf7* becomes more dominant (7 *irf7*- vs 3 *irf3*-dominant genes). By the late phase, *irf7* is clearly dominant (8 *irf7*- vs 2 *irf3*-dominant genes). This progression demonstrates the dynamic way in which *irf3* and *irf7* coordinate immune defense with *irf3* as key for early immune responses and *irf7* driving sustained inflammatory amplification and adaptive immune coordination.

Beyond *irf3* and *irf7*, our findings need to be viewed in the broader context of the interferon regulatory factor (IRF) family and the diverse immune cell types that participate in antiviral responses. Several IRFs are expressed in a widespread manner across tissues, including IRF1, IRF2, *irf3* and IRF9/p48, whereas others such as IRF4, *irf7* and IRF8 show a more restricted pattern and are preferentially enriched in cells of the immune system 41-43. *Irf3*, IRF5, *irf7* and IRF9 are central components of the type I interferon axis and are required for robust IFN-α/β production and the activation of antiviral defence programmes, while IRF1, IRF4 and IRF8 are particularly important for initiating and shaping antigen-specific immune responses 43-46. For example, IRF1 and IRF8 are indispensable for the development of Th1 immunity, in part because IL-12 production, the key cytokine that drives Th1 differentiation, is impaired in Irf1-/- and Irf8-/- mice 46, 47. IRF4 is constitutively expressed in B cells and is strongly induced in T cells upon TCR stimulation, and gene-targeting studies have shown that loss of IRF4 leads to combined B-cell and T-cell defects 48. In parallel, IRF5 has been implicated in promoting pro-inflammatory cytokine expression and in driving macrophage polarization toward inflammatory phenotypes 49.

These prior studies underscore that multiple IRF family members are likely to intersect with the* irf3*- and *irf7*-driven programmes that we delineate in alveolar macrophages and conventional dendritic cell subsets. Plasmacytoid dendritic cells, NK cells, effector and memory T cells, B cells and non-myeloid lung stromal cells each integrate distinct combinations of IRF activities during viral infection, and the global *Irf3-/-* and *Irf7-/-* models used here inevitably capture the net effect of these multilayered networks at the organismal level. Our NanoString, single-cell and NMF-based analyses suggest that the *irf3*/*irf7*-dependent modules we observe in AM, DC1 and DC2 overlap with pathways that have been linked to IRF1, IRF4, IRF5 and IRF8 in previous work, including antigen presentation, IL-12-driven Th1 priming, cytokine production and inflammatory signaling. A systematic, multi-omics dissection of IRF1/3/4/5/7/8 activity across broader immune cell populations and across different respiratory viruses will therefore be an important direction for future studies and will help to place the *irf3*-*irf7* balance described here within the full regulatory landscape of the IRF family.

A limitation of the *irf3*-deficient mouse model is that the *irf3* knockout allele also disrupts the neighboring *Bcl2l12* gene, leading to concurrent Bcl2l12 deficiency 50. We recognize that the concurrent *Bcl2l12* deficiency in the *irf3^-/-^* mouse model, represents a significant potential confound to our *in vivo* findings. *Bcl2l12* is known to regulate apoptosis, a process fundamentally intertwined with viral pathogenesis and the shaping of immune responses 51. The *Irf3* knockout allele's concomitant disruption of Bcl2l12 may alter apoptotic thresholds in specific cell types, which could influence immune cell survival and tissue pathology during influenza infection, as suggested by previous work 52. Because both *irf3-/-* and* irf7-/-* lines are global, whole-body knockout models, all *in vivo* phenotypes observed here, including survival, weight loss, BALF cytokines and lung immune-cell composition, reflect the combined contributions of hematopoietic and non-hematopoietic compartments. This limitation is acknowledged, and future studies using conditional, lineage-restricted *Irf3* and *Irf7* alleles will be required to dissect cell type-specific roles in macrophages and dendritic cells. However, multiple lines of evidence indicate that the loss of *irf3* predominantly accounts for the core phenotypes observed, particularly the altered interferon and cytokine transcriptional programs. Our study's primary focus was on the direct transcriptional regulation of innate immune pathways, such as the robust *irf3*-dependency of *Ifnb1* and *Cxcl10* expression observed in our *in vitro* stimulation assays (**Figure 5**), where long-term apoptotic effects are minimized. This points to a direct role for *irf3* in transcriptional activation that is distinct from *Bcl2l12's* function in apoptosis. Furthermore, our scGPT computational modeling, which simulates the specific impact of *irf3* loss on gene networks, successfully predicted the downregulation of canonical interferon-stimulated genes and identified *irf3*-exclusive regulatory pathways like IL-17 signaling. The consistency between these *in silico* predictions and our experimental data reinforces the conclusion that a primary *irf3*-dependent signaling defect, rather than a general apoptotic dysregulation, underlies our key findings. Nonetheless, we cannot completely rule out a contribution from *Bcl2l12* deficiency to the overall *in vivo* phenotype, especially concerning immune cell population dynamics and long-term pathogenesis. Future studies employing alternative models, such as conditional *irf3* knockout mice or models where the *Bcl2l12* gene is specifically restored, will be crucial to definitively dissect the distinct contributions of these two neighboring genes to antiviral immunity.

Moreover, our short-term *in vitro* experiments using BMMO and dendritic cells are designed to capture early transcriptional responses and are therefore less likely to be substantially influenced by altered apoptotic programs, prolonged *in vivo* influenza infection extends over many days and may be sensitive to changes in apoptotic thresholds. In this context, altered survival of alveolar macrophages, dendritic cell subsets or other immune and non-immune populations could contribute to the observed recovery-phase phenotypes in *irf3*^-/-^ mice. Accordingly, the *in vivo* effects associated with *irf3* deficiency should be interpreted as the combined consequence of *irf3* loss and *Bcl2l12* disruption. Definitive separation of these contributions will require future studies using *Irf3* knockout models that preserve *Bcl2l12* expression or conditional, lineage-restricted *irf3* deletions integrated with direct assessment of cell survival and apoptosis during infection.

Our findings on *irf3* deficiency protecting against IAV-induced lethality are opposite to Chakravarty *et al.* 53, who reported that macrophage-specific *irf3* deletion exacerbates lung inflammation in Sendai virus models. Chakravarty *et al.* noted higher mortality with Sendai virus due to unchecked inflammation, whereas our global *irf3*^-/-^ mice showed mitigated morbidity and faster recovery against IAV, likely due to compensatory mechanisms in non-myeloid cells and distinct viral signaling (IAV's TLR3/7 versus Sendai's RIG-I pathways). Both studies highlight *irf7*'s role in driving antiviral responses in AM and DC2, suggesting synergy. In line with our observations, O'Connor and Green also reported that *irf3*-deficient mice exhibited increased resistance to retrovirus-induced disease, whereas *irf7*-deficient mice remained as susceptible as wild-type controls 54. Despite the caveat of background strain contamination in their model, the overall conclusion that *irf3* loss confers relative resistance to disease progression while *irf7* loss does not alter susceptibility is consistent with our findings in IAV infection. Together, these studies reinforce a paradoxical role for *irf3*, where its deficiency can mitigate viral pathogenesis, while underscoring *irf7*'s dominant function as the principal driver of antiviral programs in alveolar macrophages and DC2 subsets.

Macrophages and DCs exhibit distinct cytokine, type I interferon and ISG expression profiles in response to TLR3 and TLR7 stimulation. Our results are consistent with previous studies showing that macrophages and DCs have distinct sensitivities to TLR agonists 31, 55, 56. Specifically, macrophages display heightened sensitivity to Poly(I:C) (dsRNA) and DCs respond more strongly to R848 (TLR7 agonist), suggesting specialization in pathogen recognition that likely reflects their distinct roles in the immune response. This specialization may be attributed to the differential expression of TLRs and their associated adaptor molecules in these cell types 57, 58. For instance, macrophages may express higher expression of TLR3 or TRIF, explaining their robust response to Poly(I:C), while DCs may respond better to R848, which might be due to enhanced expression of MyD88 or TLR7 55. The differential regulation of *irf3* and *irf7* in these cell types is particularly significant. Sustained upregulation of *irf3* in wild-type cells following Poly(I:C) treatment, with minimal changes in MyD88^-/-^ cells, suggests that MyD88 may be required for optimal *irf3*-mediated responses to dsRNA. This finding extends our understanding of the regulatory networks governing *irf3* and *irf7* activation 4, 7, 59 and identifies a previously unappreciated role for MyD88 in *irf3* regulation.

We also acknowledge that the use of global knockout mice limits our ability to strictly isolate myeloid-specific effects in vivo. Because Irf3 is broadly expressed and Irf7 is immune-enriched but highly inducible in both hematopoietic and non-hematopoietic compartments, their absence in non-hematopoietic cells, particularly lung epithelial cells and fibroblasts, could contribute to the observed differences in survival and pathogenesis 1, 60. Lung epithelial cells are the primary site of IAV replication and a critical source of type I interferons and pro-inflammatory cytokines that initiate the antiviral response 32, 61. Consequently, the protective phenotype observed in *Irf7*^-/-^ mice may partly arise from altered viral sensing or cytokine production within the structural compartment 3, 62. At the same time, our *in vitro* assays using BMMO and dendritic cells, together with transcriptomic profiling of sorted alveolar macrophages and conventional DC subsets, were designed to minimize systemic and non-myeloid influences and to focus on cell-intrinsic transcriptional responses. These findings are further supported by re-analysis of public single-cell RNA-seq and spatial transcriptomics datasets, which independently recapitulate IRF3- and IRF7-associated transcriptional programs in myeloid populations across infection contexts. These complementary approaches are consistent with the interpretation that IRF3 and IRF7 drive distinct, myeloid-intrinsic regulatory programs in macrophages and dendritic cells under viral stimulation. Nevertheless, we recognize that definitive partitioning of immune versus non-immune contributions *in vivo* will require future studies employing lineage-specific conditional knockout strategies, such as LysM-Cre or CD11c-Cre-mediated deletion, to fully disentangle compartment-specific roles in influenza pathogenesis.

Cell type-specific and stimulus-dependent nuclear translocation of *irf3*, *irf7*, and p65 provides mechanistic insight into the differential responses observed in BMMOs and BMDCs. The predominant translocation of *irf3* and p65 to the nucleus following Poly(I:C) in dendritic cells vs the inhibition of p65 nuclear translocation in DC suggest distinct cell-type pathogen recognition and signaling mechanisms. These findings are consistent with previous reports on the differential activation of IRFs in various cell types 37, 63 and highlight the importance of considering cell type-specific responses when studying innate immune signaling. The differential translocation patterns observed may be attributed to varying levels of upstream signaling molecules or differences in post-translational modifications of IRFs between cell types 64. Furthermore, these distinct patterns could lead to the activation of different sets of target genes, contributing to the specialized functions of macrophages and DCs in the immune response 4, 7.

Our multi-omics approach, combining bulk RNA-seq, NanoString analysis, and single-cell transcriptomics, provides a comprehensive view of the immune landscape during influenza infection. Distinct clustering patterns observed in the PCA analysis and identification of cell type-specific gene signatures reveal the complex interplay between genotype and cell type in shaping the transcriptional response to infection. KEGG pathway enrichment analysis revealed distinct gene regulation patterns when *irf3* or *irf7* is deficient, highlighting the diverse roles of *irf3* and *irf7* in different cell types. We also observed enhanced phagocytic activity and antigen presentation pathways in knockout cells suggesting the existence of compensatory regulatory mechanisms that maintain crucial immune functions in the absence of these transcription factors 65. This compensatory mechanism could involve the upregulation of other IRF family members or the activation of alternative signaling pathways 63. Single-cell transcriptomics analysis further uncovered significant shifts in the cellular composition of lung tissue following influenza virus infection. We observed an increase in CD11b+AM and decrease in CD206+AM subpopulations, which may have significant implications for the regulation of inflammatory responses and tissue repair in the infected lung 3, 66. These results are consistent with recent studies highlighting the plasticity of AM during respiratory infections and their crucial role in maintaining lung homeostasis.

Our study provides novel insights into the cell type-specific roles of *irf3* and *irf7* in innate immune responses and viral infection control. The findings regarding their function in influenza pathogenesis highlight the need for a nuanced understanding of these transcription factors in different cellular contexts. These results have potential implications for the development of targeted therapies for viral infections, possibly through the modulation of *irf3* and *irf7* activity in specific cell types. Future research should aim to clarify the mechanisms underlying the compensatory immune pathways activated in the absence of *irf3* and *irf7*. This could involve a more detailed analysis of other IRF family members and their interactions with various signaling pathways. Consistent with our observations of *irf7*'s dominant role in antiviral programs, human patients with autosomal recessive *irf7* deficiency exhibit selective vulnerability to respiratory viral infections, including IAV and SARS-CoV-2, while remaining otherwise healthy due to residual IFN-β production and expanded virus-specific memory T cells 67. This highlights potential compensatory mechanisms in *irf7* deficiency that warrant further investigation in both mouse models and human cohorts.

Additionally, investigating the long-term effects of *irf3* and *irf7* deficiency on immune memory and susceptibility to subsequent infections would deepen our understanding of their roles in shaping adaptive immunity. The therapeutic applications of our findings should also be explored, such as examining whether cell-type specific inhibition of *irf3* or *irf7* can be used as a strategy to modulate the immune response during viral infections 68. Although our cross-species analyses reveal broad patterns of conservation in *irf3*- and *irf7*-associated transcriptional modules between murine and human myeloid cells, these findings should be interpreted as indicative rather than definitive. The comparisons rely on public single-cell datasets and computational alignment, rather than on patient-matched influenza lung tissue, and therefore provide supportive evidence for conserved regulatory logic rather than direct validation in human disease. The absence of bronchoalveolar lavage or spatially resolved samples from individuals with varying severity of influenza thus represents an important limitation of the present study. Nevertheless, recent human genetic data underscore the clinical relevance of *irf7*-centered antiviral immunity 67. Individuals with autosomal recessive *irf7* deficiency, despite being otherwise healthy and capable of mounting robust influenza- and SARS-CoV-2-specific memory T cell responses, are predisposed to severe viral pneumonia caused by influenza virus, SARS-CoV-2, respiratory syncytial virus and adenovirus. In these patients, peripheral cells produce minimal type I and III interferons, with IFN-β serving as the major remaining antiviral signal 67. The broad age distribution at disease onset and evidence of prior exposure to common viruses suggest that compromised early innate antiviral defense, rather than global immune impairment, underlies this phenotype. This clinical presentation is consistent with the IRF7-dominant programs identified in our murine data and supports the biological importance of IRF7 in human antiviral responses. Future studies incorporating single-cell or spatial profiling of human influenza lung samples across disease severities will be critical to determine whether the *IRF3*-IRF7 balance we describe in murine alveolar macrophages and dendritic cells similarly governs antiviral immunity and pathological outcomes in human respiratory infections. Although our in vivo experiments provide valuable insights, our mouse models may not fully recapitulate the complexities of human immune responses. Furthermore, our analysis focused on specific time points and cell types, and a more comprehensive temporal and spatial analysis could reveal additional nuances in the roles of *irf3* and *irf7*.

In conclusion, our study advances the understanding of the cell type-specific functions of *irf3* and *irf7* in innate immunity and viral infection control. It highlights the complexity of innate immune regulation and opens new avenues for research into targeted immunomodulatory therapies. Future studies building on these findings have the potential to refine our approaches to treating viral infections and immune-related disorders.

## Materials and Methods

### Mice

C57BL6 mice were obtained from the National University of Singapore Centre for Animal Resources. *TLR^-/-^* BMMO and BMDCs on C57BL/6 background were provided by our collaborator Dr. AM. Fairhurst from the Singapore Immunology Network. *Irf3*-/- and *irf7-/-* mice used in this study were obtained from the RIKEN BioResource Center. The IRF3-deficient strain (B6;129S6-Bcl2l12/Irf3<tm1Ttg>/TtgRbrc; RBRC00858) is a semi-congenic C57BL/6 × 129S6 line generated using CCE/EK.CCE ES cells derived from 129S/SvEv. In this model, a 2.2-kb genomic region of *Irf3* containing the transcription initiation site and the N-terminal DNA-binding domain was replaced, resulting in complete loss of IRF3 protein expression. Importantly, the targeted deletion spans the neighboring Bcl2l12 locus, leading to concomitant *Bcl2l12* deficiency. As *Bcl2l12* encodes a pro-apoptotic Bcl-2 family protein, this dual disruption may influence apoptosis and tissue repair during viral infection and should be considered when interpreting *in vivo* phenotypes 52.

The *irf7*-deficient strain (B6;129P2-Irf7<tm1Ttg>/TtgRbrc) is a semi-congenic C57BL/6 × 129P2/OlaHsd line generated using E14.1 ES cells. In this allele, exons 2 and 3 of the *Irf7* gene were replaced with a PGK-β-geo cassette, generating a null mutation with complete loss of *irf7* expression. Homozygous *irf7*-/- mice exhibit markedly reduced type I interferon responses and increased susceptibility to viral infection. All knockout strains were maintained by homozygote × homozygote breeding and backcrossed for multiple generations into C57BL/6J in our facility. All animal work was approved by the Institutional Animal Care and Use Committee and followed National Advisory Committee for Laboratory Animals Research (NACLAR) Guidelines (Guidelines on the Care and Use of Animals for Scientific Purposes).

### Viruses

Mice were transferred to the ABSL2 facility for experiments involving infection with IAV. Mice were infected intra-tracheally with 500 pfu of H1N1/PR8 virus in 20 μl of PBS. Their body weight was measured daily and they were euthanized when a loss of 15-20% of initial body weight was observed. Bronchoalveolar lavage fluid was obtained from uninfected and infected mice at specific days post-infection. Fluids were spun down to separate leukocytes and supernatants were analyzed for cytokines. Leukocytes were stained with CD45, CD11c, CD3/B220, Ly6G and Siglec F to determine the cellular composition using flow cytometry. Eosinophils-CD45+ Siglec F+ Ly6G+ F4/80- CD11clow; Neutrophils (PMNs)-CD45+ Siglec F- Ly6G+; Alveolar macrophages-CD45+ Siglec F+ Ly6Gint CD11c+ F4/80+; Lymphocytes -CD45+ Siglec F- Ly6G- F4/80- CD14- CD3/CD19+.

### Isolation and *In Vitro* Stimulation of Macrophages and DCs

Bone marrow was extracted using a protocol described previously 69. BMMO and BMDCs were cultured from isolated bone marrow cells by incubating with M-CSF and GM-CSF respectively for seven days. Treatment of BMMO and BMDCs were done using 100ng/mL R848 and 10μg/mL poly(I:C) as required.

### RNA Purification and qPCR

RNA purification and quantitative real-time polymerase chain reaction (qPCR) were carried out as described previously 70. qPCR was carried out using the Applied Biosystems 7900 Fast Real-Time PCR system, and expression levels were analysed using the 2^-ΔΔCT^ method with GAPDH as the endogenous control. Primers used to amplify DNA sequences: Mouse IL-6 (F) 5'-GGGACTGATGCTGGTGACAA-3' (R) 5'-TCCA CGATTTCCCAGAGAACA-3'; Mouse TNF-α (F) 5'-GGCAAGGATGAGCCTTTTAGG-3' (R) 5'-TTGGTTTGGGAGGAAAGGG-3'; Mouse CXCL10 (F) 5'-GGACGGTCCGCTGCAA-3' (R) 5'-GCTTCCCTATGGCCCTCATT-3'; Mouse IFN-α (F) 5'-TGTCTGATGCAGCAGGTG-3' (R) 5'-AAGACAGGGCTCTCCAGA-3' Mouse IFNβ (F) 5'-GGCGGACTTCAAGATCCCTAT 3'; (R) 5'GGATGGCAAAGGCAGTGTAAC-3'.

### Enzyme-Linked Immunosorbent Assay

Cytokine concentrations in culture supernatants were measured using Ready-Set-Go® ELISA kits (eBioscience), following the manufacturer's instructions. Briefly, 96-well plates were coated with capture antibodies overnight, followed by blocking and incubation with samples or standards. After washing, detection antibodies and streptavidin-HRP were applied sequentially, and the signal was developed using TMB substrate and quantified by absorbance at 450 nm using a microplate reader.

### Immunofluorescence and Microscopy

Cells were seeded onto sterilized glass coverslips in a 24-well plate and treated with the respective TLR agonists for one hour, before staining with rabbit anti-IRF3 or rabbit anti-p65 antibodies (Cell Signalling Technology) diluted in 2% BSA (v/v), 2% FBS (w/v) in PBS. The coverslip was then washed before incubation with a fluorophore-conjugated secondary Ab Alexa 488 anti-rabbit IgG (Invitrogen), followed by mounting and imaging using a Zeiss 510 Meta System laser scanning microscope. Images were processed using the ImageJ software.

### Bulk RNA Sequencing Analysis

The RNA sequencing dataset (**GSE124404**), obtained from mice of six blood samples included three control mice and three mice with influenza (A/PR/8) at 48 hours post-infection (hpi), were used for differentially expressed genes (DEGs) analysis. The DEGs were selected based on log fold change (LogFC) and p-value using the R package DESeq2 (v1.48.1). Additionally, the R package immunedeconv (v2.1.3) was employed for deconvolution analysis to estimate immune cell fractions and ratios.

### NanoString Gene Expression Analysis

NanoString data from wild type (WT), *irf3* knockout (KO), and *irf7* KO alveolar macrophages (AM), DC subset 1 (DC1), and DC subset 2 (DC2) under influenza infection and uninfected conditions were analyzed. Differential expression analysis was performed using DESeq2 (v1.48.1) in R (v4.3.1). Genes with an adjusted p-value < 0.05 and |log2FoldChange| > 1 were considered differentially expressed.

### Principal Component Analysis

Principal Component Analysis was performed on the regularized log-transformed count data using the prcomp function in R. The top 500 most variable genes across all samples were used for the PCA. Results were visualized using ggplot2 (v3.5.2), with the first two principal components plotted and sample groups indicated by different colors and shapes.

### KEGG Pathway Enrichment Analysis

KEGG pathway enrichment analysis was conducted using the clusterProfiler package (v4.17.0) in R. For each condition (WT, *irf3* KO, *irf7* KO) and cell type (AM, DC1, DC2), separate analyses were performed for up- and down-regulated genes. The universe of genes was set to all protein-coding genes detected in the RNA-seq data. Pathways with an adjusted p-value < 0.05 were considered significantly enriched. The top 5 most significantly enriched pathways (based on adjusted p-value) for each condition were selected for visualization. Visualization The KEGG pathway enrichment results were visualized using ggplot2. The plot was customized using theme_classic() and additional theme modifications for improved readability. All statistical analyses and visualizations were performed in R (v4.3.1).

### Single-Cell RNA Sequencing analysis

Single-cell analysis was conducted using datasets for influenza-infected mice (GSE228594), COVID-19 (GSE201266), HSV-infected mice (GSE161336), and Sendai virus-infected mice (GSE178517). Downstream analysis of the influenza dataset (GSE228594) was performed using the Seurat R package (v4.3). Cells were first filtered for quality control by retaining cells with 500-5,000 detected genes and < 10% mitochondrial transcripts. Gene expression was then normalized and variance-stabilized using SCTransform with default settings, followed by principal component analysis (PCA). Unsupervised clustering was carried out using a shared-nearest-neighbor graph and the Louvain algorithm at resolutions between 0.4 and 0.8, and UMAP was employed for dimensional reduction and visualization. Cell types were annotated based on the expression of established marker genes for CD206+ alveolar macrophages (AM), CD11b+ AM, DC1, DC2 and T cells. Where multiple samples or batches were present, SCTransform-based integration was used to correct batch effects prior to clustering and visualization. Differential abundance and differential expression analyses between control and influenza-infected samples were performed using the FindMarkers function in Seurat with default statistical tests. Single-cell datasets from other viral infections (COVID-19, HSV, Sendai virus) were processed analogously, applying the same quality control thresholds, normalization procedure and clustering strategy, unless otherwise stated.

### Spatial Data Analysis

Downstream spatial analysis data from dataset (GSE202322) was performed using the Seurat R package (v4.3). Quality control was performed to filter out low-quality data points; specifically, spots with fewer than 500 detected genes were excluded from downstream analysis to minimize technical noise. Normalization and variance stabilization were conducted using the SCTransform method to account for sequencing depth and technical variation. Dimensionality reduction was performed using Principal Component Analysis (PCA) on the top variable features. To identify distinct tissue domains, unsupervised clustering was applied to the top principal components using a graph-based clustering approach (Louvain algorithm). Visualization of gene expression patterns and spatial domains was generated using the SpatialFeaturePlot function, while expression distributions were assessed using violin plots. Differential expression analysis was performed using the FindMarkers function with a Wilcoxon rank-sum test. Genes exhibiting a Benjamini-Hochberg adjusted p-value < 0.05 were considered significantly differentially expressed.

### Trajectory and Cell-Cell Communication Analysis

Single-cell trajectories were constructed using Monocle3 (v.1.4.26). After preprocessing and quality control, cells were dimensionally reduced using UMAP. The trajectory graph was constructed using the learn_graph function with default parameters, and cells were ordered along the trajectory using order_cells. Pseudotime values were calculated relative to the manually selected root state based on monocyte markers. Cell-cell communication analysis was performed using CellChat (v.1.5.0). Expression data were preprocessed and normalized following standard workflow. Ligand-receptor interactions were identified using CellChat's mouse database. Significant interactions were determined using default statistical methods with permutation test (p < 0.05). Communication patterns were analyzed at both individual interaction and pathway levels. Interaction strength was quantified using probability scores, and communication networks were visualized using circle plots and heatmaps. Differential cell-cell communication analysis between conditions was performed using the *compareInteraction*s function.

### Statistical Analysis

Statistical analyses were performed using GraphPad Prism v10 and R v4.3.1. Data were expressed as mean ± SEM unless otherwise stated. For comparisons involving more than two groups, one-way or two-way ANOVA was used followed by Tukey's or Dunnett's multiple comparison tests. For two-group comparisons, unpaired two-tailed Student's t-test was applied. A *p-value* of less than 0.05 was considered statistically significant. Where applicable, results were derived from at least three independent experiments with biological replicates. Significance levels are indicated in figure legends.

## Supplementary Material

Supplementary figures and tables.

## Abbreviations

AM: alveolar macrophages
BAL: bronchoalveolar lavage
BMDC: bone marrow-derived DCs
BMMO: bone marrow-derived macrophages
BSA: bovine serum albumin
CCL: C-C motif chemokine ligand
cDCs: conventional DCs
CXCL: C-X-C motif chemokine ligand
DAPI: 4',6-diamidino-2-phenylindole
DC1: DC subset 1
DC2: DC subset 2
DEG: differentially expressed genes
FACS: fluorescence-activated cell sorting
GM-CSF: granulocyte-macrophage colony-stimulating factor
GO: gene ontology
GSVA: gene set variation analysis
HRP: horseradish peroxidase
IAV: influenza A virus
IFN: interferon
IFN-I: type I interferon
IL: interleukin
IRF: interferon regulatory factor
ISG: interferon-stimulated gene
JAK-STAT: Janus kinase-signal transducer and activator of transcription
KEGG: Kyoto Encyclopedia of Genes and Genomes
KO: knockout
M-CSF: macrophage colony-stimulating factor
MyD88: myeloid differentiation primary response 88
NF-κB: nuclear factor kappa B
PCA: principal component analysis
pDCs: plasmacytoid DCs
PFU: plaque-forming units
PI3K: phosphoinositide 3-kinase
PMN: polymorphonuclear neutrophils
Poly(I:C): polyinosinic:polycytidylic acid
PRR: pattern recognition receptor
qPCR: quantitative polymerase chain reaction
RMSE: root mean square error
RNA-seq: RNA sequencing
scGPT: single-cell generative pre-trained transformer
SDS-PAGE: sodium dodecyl sulfate-polyacrylamide gel electrophoresis
STING: stimulator of interferon genes
TBK1: TANK-binding kinase 1
TLR: Toll-like receptor
TMB: 3,3',5,5'-tetramethylbenzidine
TNF: tumor necrosis factor
TRIF: TIR-domain-containing adapter-inducing interferon-β
UMAP: uniform manifold approximation and projection
WT: wild-type
