# Supplementary material for: Irf7 Deficiency Confers Protection Against Influenza Infection, Independent of irf3
Source: Int J Biol Sci. 2026 Jan 22;22(4):1974–96. doi: 10.7150/ijbs.126714 (PMC12905640; doi:10.7150/ijbs.126714)
Supplement: Supplementary file 1 — Supplementary figures and tables. [file ijbsv22p1974s1.zip › Final_version_ IRF37_Supplementary M_M_08_Jan_2026.docx]

# **Supplementary Materials and Methods**

*Irf7* Deficiency Confers Protection Against Influenza Infection, Independent of *irf3*

Jianzhou Cui^1, 2, 3, 4 †^, Sherman S. W. Foo^1,2,3^ ^†^, Wan Ting Kong^1,2,3^, Chenshi Lin^1,3,4^, Patrick B. Ampomah^1,2,3^, Olga Zharkova ^1,2,3^, Loo Soon Chai^1,2,3^, Karishma Sachaphibulkij^1,2,3,4^, Suruchi Arora^1,2,3^, Nivashini Kaliaperumal^5^, Hong Meng Lim^1,2,3^, John Connolly^5^, Anna-Marie Fairhurst^5,6^, Jinmiao Chen^1,7,8^, Lina H. K. Lim^1,2,3, #^

^1^Immunology Translational Research Program, Yong Loo Lin School of Medicine, National University of Singapore, Singapore, Singapore

^2^Department of Physiology, Yong Loo Lin School of Medicine, National University of Singapore, Singapore, Singapore

^3^Immunology Program, Life Sciences Institute, National University of Singapore, Singapore, Singapore

^4^NUS-Cambridge Immunophenotyping centre, National University of Singapore, Singapore, Singapore

^5^Institute of Molecular and Cellular Biology (IMCB), Agency for Science, Technology and Research, Singapore, Singapore

^6^Singapore Immunology Network (SIgN), Agency for Science, Technology and Research, Singapore, Singapore

^7^Bioinformatics Institute (BII), Agency for Science, Technology and Research, Singapore, Singapore

^8^Center for Computational Biology and Program in Cancer and Stem Cell Biology, Duke-NUS Medical School, Singapore, Singapore

^†^These authors contributed equally

**Correspondence:**

^#^Dr. Lina H.K. Lim,

Department of Physiology, Yong Loo Lin School of Medicine, National University of Singapore

Immunology Program, Yong Loo Lin School of Medicine, National University of Singapore, Life Science Institute Level 3, 28 Medical Drive, Singapore 117456, [linalim@nus.edu.sg](mailto:linalim@nus.edu.sg)

## **Supplementary Methods**

### **1 Non-negative Matrix Factorization (NMF) analysis**

NMF was performed using the NMF package in R. To select the factor number (rank), we evaluated a candidate range of ranks and compared model stability and reconstruction quality metrics, including cophenetic correlation–based stability, dispersion, and residual approximation error. Rank = 6 was chosen as a parsimonious optimum that achieved stable solutions while avoiding over-fragmentation into redundant factors.The NMF algorithm was applied to the normalized gene expression matrix to decompose it into two non-negative matrices: a basis matrix (W) representing the gene expression programs (factors) and a mixture matrix (H) representing the activity of these factors in each sample. The seed argument was set for reproducibility.

Based on gene ontology and KEGG pathway enrichment analysis of factor-associated gene signatures, we functionally annotated these **six factors:** Factor 1 - "Proliferative & Inflammatory Signaling" (enriched for immune effector process regulation, lymphocyte proliferation, and cytokine-mediated signaling path-ways, with key genes including *H2-K1, Ccl2, Il1b, and Irf7*); Factor 2 - "Antigen Presentation & T Cell Activation" (dominated by antigen processing/presentation and lymphocyte activation programs, featur-ing genes like *Cd74, H2-Aa, H2-Ab1, and Cd83*); Factor 3 - "Negative Regulation of Cell Differentiation & Peptidase Activity" (characterized by regulation of cysteine-type endopeptidase activity and negative regulation of hematopoiesis, with genes such as *Vegfa, Myc, and Il4ra*); Factor 4 - "Macrophage-Associated Inflammatory Response" (enriched for chemokine and TNF production regulation, featuring characteristic genes like *Chil3, Chil4, and Sirpa*); Factor 5 - "Adaptive Immune Activation & Cell Adhe-sion" (combining antigen presentation machinery and leukocyte adhesion pathways, with key genes in-cluding *H2-Aa, Cd74, Ccl5, and Cd83*); and Factor 6 - "Adhesion & Antigen Presentation" (featuring cell-cell adhesion and MHC-related functions with genes like *Cd74, H2-Aa, and Klf4*)

For each identified NMF factor, the top 30 genes contributing most significantly to that factor (i.e., genes with the highest weights in the W matrix for that factor) were selected. Gene Ontology (GO) biological process enrichment analysis was performed on these gene lists using the clusterProfiler package (v3.18.1) in R with org.Mm.eg.db as the organism database for mouse genes. Pathways with an adjusted p-value (Benjamini-Hochberg corrected) less than 0.05 were considered significantly enriched. The top 5 enriched GO terms for each factor were used for functional annotation and naming of the factors. The sample-specific activities of the NMF factors (H matrix) were visualized using a heatmap generated with the ComplexHeatmap package in R. The factor activities were scaled (e.g., Z-score normalization) to improve visualization of relative differences. Samples were annotated by Cell_Type, Treatment, Genotype, and Gene_Type using distinct colors to highlight the experimental conditions. Clustering was applied to both rows (factors) and columns (samples) to group similar patterns. A Sankey diagram was generated to illustrate the connections between the NMF factors, their most representative genes, and the enriched GO biological processes. This visualization was created by integrating the top genes per factor and the enrichment analysis results, using a suitable R package for Sankey diagrams (networkD3 and ggalluvial).

### **2 Quantification of IRF Regulatory Activity (IRF-RAS)**

To quantify the regulatory activity of IRF3 and IRF7 at the single-cell level, we developed an IRF Regulatory Activity Score (IRF-RAS) framework. Putative target genes for IRF3 and IRF7 were curated based on published literature, the ChEA3 database, and regulons inferred by SCENIC+.
Single-cell gene set enrichment scores were computed using the AUCell algorithm on normalized expression matrices. For each cell, three metrics were calculated:

- **IRF3 Score:** AUCell enrichment score for the IRF3 target gene set.
- **IRF7 Score:** AUCell enrichment score for the IRF7 target gene set.
- **IRF-Balance Index:** A normalized index reflecting the relative dominance of IRF3 or IRF7 activity, computed as:

$$IRF-Balance Index = (IRF7 Score - IRF3 Score) / (IRF7 Score + IRF3 Score)$$

### **3 scGPT framework and analysis results**

#### 3.1 Fine-Tuning of scGPT on Mouse Lung scRNA-seq Data

The scGPT framework can be found in **Figure S4A.**

**Step 1: Training objective and dataset composition.** Single-cell RNA-sequencing (scRNA-seq) data were obtained from influenza-infected mouse lungs. After preprocessing with Scanpy (v1.9.6), five immune cell types were annotated: CD206⁺ alveolar macrophages (AM), CD11b⁺ AM, dendritic cells subtype 1 (DC1), dendritic cells subtype 2 (DC2), and T cells. The scGPT model was fine-tuned to learn cell-type-resolved transcriptional structure in our mouse lung immune compartment, using WT cells only as the training set (n = 1,852) spanning five annotated populations. The fine-tuning objective was to optimize latent representations that preserve immune cell identity and gene–gene dependencies in this dataset, providing a calibrated in-domain model for downstream conditional simulation. The gene expression matrix was log-normalized and saved in .h5ad format.

**Step 2: Vocabulary Construction and Model Initialization** A gene vocabulary was built from the union of genes in all WT cells. A special <pad> token was assigned index 0 to allow for padded sequences. The TransformerModel was initialized with 512-dimensional embeddings, 8 attention heads, 6 encoder layers, and a hidden dimension of 2048. Token sequences and corresponding continuous expression values were provided as model inputs.

**Step 3: Model architecture and optimization hyperparameters.** The TransformerModel was initialized with 512-dimensional gene embeddings, 8 attention heads, 6 encoder layers, and a 2048 hidden dimension. Training was performed using PyTorch Lightning (v2.2.0) on CPU with the AdamW optimizer (learning rate = 1×10⁻⁴), batch size = 64, and 10 epochs. Categorical cross-entropy was used for the cell-type supervision component, and model checkpoints were saved based on minimum training loss. A fixed random seed was used to ensure reproducibility of training and inference outputs.

**Step 4: IAV-specific conditioning and perturbation formulation.** “IAV-specific perturbation simulation” in this study refers to conditioning inference on infection status (Flu vs Control) within the prompt, rather than training separate infection-specific models. After the WT in-domain fine-tuning, we constructed structured prompts consisting of cell_type, infection state (Flu/Control), and genotype/perturbation state (IRF3-KO, IRF7-KO, None), and generated virtual expression outputs from the model. In this formulation, the infection label is treated as a conditioning context to query the model for infection-state-dependent transcriptional programs under each KO. For each condition, 1 virtual cell was simulated using token inputs and randomly initialized embedding vectors. Expression outputs were collected from the model’s masked language modeling (MLM) head. In total, 30 conditions (5 cell types × 3 KO states × 2 infection states) were simulated.

**Step 5: Nanostring Data Processing** Nanostring nCounter expression profiles were obtained for AM, DC1, and DC2 sorted from WT, IRF3-KO, and IRF7-KO mice with/without influenza infection. Raw expression counts (nano_all_expression_data_irf3and7.csv) were normalized by internal reference genes. Metadata (nano_all_metadata_irf3and7.csv) were used to map each sample to cell type, genotype, and infection state. DEGs were computed using two-sided Welch’s t-tests and adjusted via Benjamini-Hochberg correction.

**Step 6: Bulk RNA-seq DEG Integration** To validate scGPT predictions at the systemic level, we retrieved public RNA-seq data (GSE124404) from mice infected with high-dose influenza virus. DESeq2 (v1.38.3) was used to identify DEGs between flu-infected (n=3) and control (n=3) mice. Genes with FDR < 0.05 and absolute log₂ fold-change > 1 were considered differentially expressed.

**Step 7: Cross-Modality Validation and Visualization** Predicted DEGs from scGPT-generated expression matrices were compared with Nanostring and bulk RNA-seq DEGs. DEG overlaps were visualized using Venn diagrams (R package VennDiagram). Volcano plots were generated to show IRF3-KO and IRF7-KO specific perturbation effects. Additional visualizations included heatmaps, violin plots of ISGs/chemokines, and rank plots comparing predicted and observed fold changes. Predicted DEGs uniquely found in scGPT outputs were highlighted as candidate novel regulators.

**Data Availability** All simulated expression files were saved in CSV format under generated_expression/. These include 30 files corresponding to all combinations of the five cell types, three KO states, and two infection statuses.

#### **3.2 Sensitivity and robustness analyses for scGPT simulations**

**Sensitivity and robustness analyses.** To assess the robustness of scGPT-derived perturbation effects, we evaluated stability across (i) random seed variation, (ii) random initialization used during virtual cell generation, and (iii) key training hyperparameters. Specifically, we repeated inference under multiple seeds and quantified the concordance of predicted KO effects (direction and magnitude of log fold-change) for the highest-confidence genes, emphasizing reproducibility for high-expression genes where the model shows the strongest linear consistency. We additionally verified that qualitative conclusions regarding IRF3- versus IRF7-dominant programs were preserved under modest perturbations to learning rate and epoch number (within a narrow range around the primary settings). Robustness was summarized by correlation of predicted KO effect vectors and by overlap of top-ranked IRF3- and IRF7-dependent candidate genes across runs. Predictions that were unstable across seeds were not emphasized as primary mechanistic claims but were retained as exploratory hypotheses.

#### **3.3 Software and Libraries**

**Programming Languages & Frameworks**
- Python 3.9 – core programming environment for model development and downstream analysis.
- R 4.2+ – used for visualization and DEG validation (e.g., VennDiagram, ggplot2).
**Single-cell Modeling**
- scGPT (v0.2.4) – transformer-based foundation model for single-cell omics data.
- PyTorch (≥1.13) – deep learning backend for model training.
- PyTorch Lightning – high-level wrapper for PyTorch to organize training loops and checkpoints.
**Preprocessing & Data Handling**
- Scanpy – for loading, filtering, and handling .h5ad files.
- AnnData – for structured single-cell data storage.
- NumPy – matrix and array manipulations.
- Pandas – table-level data wrangling.
**Tokenizer**
- GeneVocab – custom tokenizer to convert gene names into token indices compatible with scGPT.
**Visualization & Evaluation**
- Matplotlib / Seaborn – basic plotting and customization.
- VennDiagram (R) – overlap analysis between predicted and experimental DEGs.
- ggplot2 (R) – heatmaps, volcano plots, violin plots, and rank plots.
**Others**
- torchmetrics – for model evaluation metrics (if used).
- tensorboard / CSVLogger – for training log and monitoring (optional in CPU-only runs).

#### **3.4 scGPT Simulation Consistency and Effect Magnitude Show Strong Dependence on Baseline Gene Expression Level**

The analysis revealed a striking dependence on expression level, differing from typical accuracy-vs-expression patterns. Both Pearson and Spearman correlations were near zero for genes in the lower three expression quartiles (Q1-Q3; Pearson R ≈ 0.11, 0.02, 0.00 respectively), suggesting weak concordance between baseline and KO simulations for low-to-medium abundance transcripts (**Figure S4B,** top panel). However, for the highest expression quartile (Q4), the Pearson correlation dramatically increased to ≈ 0.89, while the Spearman correlation remained low (≈ 0.13). This indicates a strong linear relationship for Q4 genes that may not preserve rank order, potentially influenced by scale effects or outliers. Concurrently, the RMSE steadily increased with expression level, rising from ≈ 0.17 in Q1 to ≈ 0.53 in Q4 (Figure S2B, bottom panel). This demonstrates that the largest absolute differences between simulated KO and baseline states occur for the most highly expressed genes. Together, these results suggest that the simulated perturbation effect captured by scGPT is most pronounced and linearly correlated with baseline primarily for the highest expression quartile, while simulations for lower expressed genes show greater divergence and weaker correlation relative to the baseline simulation.

#### **3.5 scGPT Predicts Novel IRF3- and IRF7-Regulated Gene Candidates Beyond the Nanostring Panel**

A key advantage of using scGPT is its ability to simulate expression for the entire transcriptome, allowing us to explore gene regulation beyond the scope of targeted panels like Nanostring. To identify potential novel targets, we filtered the list of genes identified as specifically regulated by IRF3 or IRF7 in our Flu simulation analysis (abs(LFC) > 0.3, specificity factor > 1.2) to exclude those present on the Nanostring panel used in our experimental validation (Methods). These remaining "novel" candidate genes were then ranked based on the maximum absolute LFC observed across the five simulated immune cell types.

The top 6 predicted novel candidates are presented in **Figure S4C** . This list highlights genes not previously assessed experimentally in our Nanostring assay but predicted by scGPT to be significantly and specifically affected by IRF3 or IRF7 knockout during influenza infection. Notably, many top candidates appeared to be IRF7-dependent, particularly in CD206+ AMs and T cells. For instance, the top-ranked gene, Ccnd2 (Cyclin D2), was predicted to be strongly downregulated specifically upon IRF7 KO simulation in CD206+ AMs (MaxAbsLFC ≈ 1.56). Other top IRF7-dependent candidates included Naaa (N-acylethanolamine acid amidase, predicted UP in CD206+ AMs), Fbxo30 (F-box protein 30, predicted DOWN in T cells), and Nampt (Nicotinamide phosphoribosyltransferase, predicted DOWN in CD206+ AMs). The analysis also identified IRF3-specific candidates, such as Cpe (Carboxypeptidase E, predicted DOWN in T cells) and Gm35330 (predicted DOWN in CD11b+ AMs). The plots generally corroborated the LFC-based ranking and specificity calls. For example, Ccnd2 expression was markedly lower specifically in the IRF7 KO simulation compared to Baseline and IRF3 KO, most prominently in CD206+ AMs. Conversely, Naaa expression increased specifically upon IRF7 KO in CD206+ AMs. Cpe showed a distinct decrease upon IRF3 KO simulation, particularly visible in T cells. These simulation results provide specific, testable hypotheses regarding novel downstream targets or modulators of IRF3 and IRF7 pathways during viral infection in distinct lung immune cells.

### **4 Temporal Dynamics Analysis and scGPT-NMF Integration Framework**

IRF3-dominant, IRF7-dominant, and co-regulated modules were defined based on scGPT knockout effect predictions. Genes with |IRF3_effect| > |IRF7_effect| were classified as IRF3-dominant, genes with |IRF7_effect| > |IRF3_effect| as IRF7-dominant, and genes with ||IRF3_effect| - |IRF7_effect|| ≤ 0.1 as co-regulated. Module functional characterization was performed using KEGG pathway enrichment analysis with Benjamini-Hochberg correction (adjusted *p < 0.05*).

Time-course data were modeled using biologically-informed regulatory pattern simulation based on known IRF3 and IRF7 activation kinetics during viral infection. Early phase (2-6 hours), middle phase (12-24 hours), and late phase (48-72 hours) classifications were based on established timelines of interferon responses. Regulation strength was quantified as the absolute log fold change multiplied by time-dependent scaling factors: early phase IRF3 genes received scaling factors of 0.8-1.2, middle phase received 0.5-0.8, and late phase 0.2-0.5, while IRF7 showed inverse temporal progression with early phase scaling of 0.1-0.3, middle phase 0.6-0.9, and late phase 0.8-1.2. The IRF3/IRF7 balance score was calculated as IRF7_regulation / IRF3_regulation, with ratios >1 indicating IRF7 dominance and ratios <1 indicating IRF3 dominance. Temporal transitions were quantified by comparing balance ratios across time points.

The integration of scGPT predictions with NMF experimental results was achieved through a multi-step correlation analysis. scGPT knockout effect predictions (IRF3 and IRF7) were mapped to corresponding NMF factor weights for each gene. Genes showing high weights in specific NMF factors were cross-referenced with scGPT predictions to identify factor-specific IRF dependencies. Validation was performed by comparing predicted regulatory relationships with experimental gene expression changes in knockout conditions.

### **5 Multi-dimensional Integration Framework**

The integration matrix combined eight data dimensions: (1) NMF factor weights (Factor 1-6) from experimental NanoString data, (2) scGPT IRF3 knockout effect predictions, (3) scGPT IRF7 knockout effect predictions, (4) spatial expression scores derived from published single-cell spatial transcriptomics data and literature-based tissue distribution knowledge, (5) temporal dynamics coefficients calculated as continuous scores from 0 (early-specific) to 1 (late-specific) based on known gene activation kinetics, (6) cell specificity indices calculated as 1 - (expression variance across cell types / maximum possible variance), and (7) clinical relevance scores derived from DisGeNET database mining and literature meta-analysis of disease associations.

**Spatial expression scores** were assigned based on known tissue distribution patterns from published single-cell atlases and immunological literature: broadly expressed genes (0.8-1.0), moderately distributed genes (0.6-0.8), and cell-type-restricted genes (0.2-0.5).**Temporal dynamics scores** were calculated based on established gene activation kinetics during viral immune responses: immediate early response genes (0.9-1.0), early-to-middle phase genes (0.7-0.9), sustained response genes (0.6-0.8), and resolution phase genes (0.4-0.6).**Cell specificity indices** were determined from published expression profiles: cell-type-specific genes (0.8-1.0), moderately specific genes (0.4-0.7), and broadly expressed immune genes (0.3-0.5). **Clinical relevance scores** were derived from systematic literature review and therapeutic database mining: well-established therapeutic targets (0.9-1.0), emerging targets (0.7-0.9), potential targets (0.5-0.7), and research-stage targets (0.2-0.5). All scoring criteria were established prior to analysis and applied consistently across the gene set to ensure objective evaluation.

Data normalization was performed using Z-score standardization for NMF factors and min-max scaling (0-1) for validation dimensions. Missing values were imputed using k-nearest neighbors (k=5) imputation for sparse measurements. Heatmap visualization was generated using ComplexHeatmap (version 2.10.0) with hierarchical clustering based on Euclidean distance metric and complete linkage method.

### 6 **Cross-Species Single-Cell Data Alignment using SAMap**

To identify transcriptionally homologous immune cell populations between mouse and human lungs during influenza infection, we employed the SAMap algorithm (version 1.0.15) . Processed single-cell RNA sequencing data from influenza-infected mouse lungs (GSE228594, focusing on CD11b+ AM, CD206+ AM, DC1, DC2, and T cell populations identified via Seurat) and influenza-infected human lungs (GSE149689, focusing on macrophage subtypes, DCs, and T cells identified via Seurat were used as input. Gene expression matrices (normalized data) and cell type annotations (celltype_sub metadata column) were retained. If necessary, Seurat objects were converted to the AnnData (.h5ad) format using the SeuratDisk R package.

A curated list of one-to-one orthologous genes between Mus musculus and Homo sapiens was obtained using the biomaRt R package querying the Ensembl database, filtering for high-confidence, one-to-one orthology. This ortholog list was provided to SAMap.

The SAMap analysis was performed using the default parameters within the SAMap Python package. Briefly, this involved mapping genes across species using the provided ortholog list, identifying mutual nearest neighbors based on conserved gene expression patterns in a shared manifold representation, and calculating mapping scores between the pre-annotated cell types from each species. The resulting mapping scores quantify the transcriptional similarity between cell populations across the two species.

We note that SAMap mapping scores can vary substantially across cell-type pairs, and relatively low scores do not necessarily indicate failure of the approach. Instead, low scores may reflect true biological divergence in infection-induced states across species, differences in cell-state granularity or annotation resolution, and constraints imposed by one-to-one ortholog mapping (particularly for immune activation modules with species-biased gene usage). Accordingly, we interpret high-scoring mappings as stronger evidence of conserved transcriptional correspondence, while low-scoring pairs are treated as suggestive and are not used for definitive one-to-one assertions. Where mapping scores were low, we emphasized convergence at the pathway/module level (e.g., interferon and inflammatory signatures) rather than strict cell-state equivalence.

### **7 Gene set variation analysis (GSVA) and pathway scoring**

Pathway activity scores were calculated using the GSVA R package (v2.2.0) with the single-sample Gene Set Enrichment Analysis (ssGSEA) method. Gene sets for IRF3- and IRF7-specific targets were defined using a conservative, consensus-based curation strategy that integrated (i) published literature-reported IRF3/IRF7 downstream targets, (ii) ChEA3 transcription factor target annotations, and (iii) IRF regulons inferred from our single-cell data using SCENIC(+), thereby reducing dependence on any single ChIP-seq dataset or prediction source. These target sets were then used for ssGSEA/GSVA scoring to quantify IRF3- and IRF7-associated pathway activity across cell types and viral conditions. . For each Seurat object representing different viral infections (influenza, SARS-CoV-2, HSV, and Sendai virus), normalized expression matrices were used as input for GSVA. The IRF balance index was calculated by subtracting the IRF3 pathway score from the IRF7 pathway score for each cell. Positive values indicate IRF7 dominance while negative values indicate IRF3 dominance. Cell-type specific pathway scores were calculated by averaging scores within annotated populations. Visualization was performed using the ggplot2 R package (v3.5.2). For gene expression analysis, cells were grouped by cell type and viral condition, and mean expression values and percentage of expressing cells were calculated for key genes in pro-inflammatory, type I IFN/ISG, and transcription factor categories.

## **Supplementary Figures**

**Figure S1 S2 S3 S4 S5**


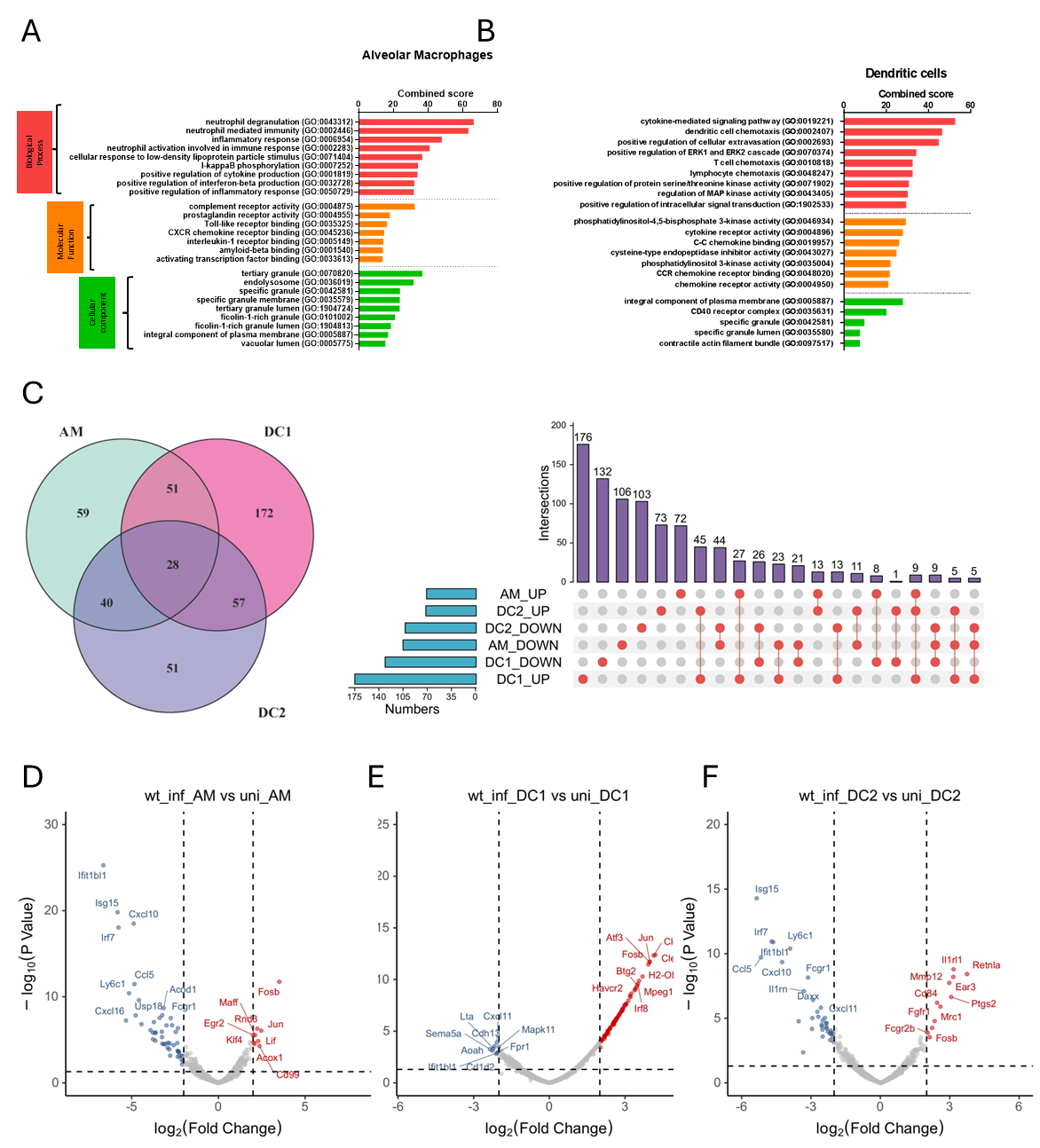


### **Figure S1 Transcriptional Profiling Reveals Distinct Functional Signatures of Lung Dendritic Cell Subsets and Alveolar Macrophages at Steady State and during Influenza Infection.**

**(A-B)** Gene ontology analysis of significantly enriched biological processes, molecular functions, and cellular components in alveolar macrophages and dendritic cells at steady state.

**(C)** Venn diagram showing the distribution and overlap of differentially expressed genes (DEGs) in AM, DC1, and DC2 in response to influenza infection.

**(D-F)** Volcano plots showing differentially expressed genes in Flu infected and specifically AM, DC1 and DC2 following influenza infection.


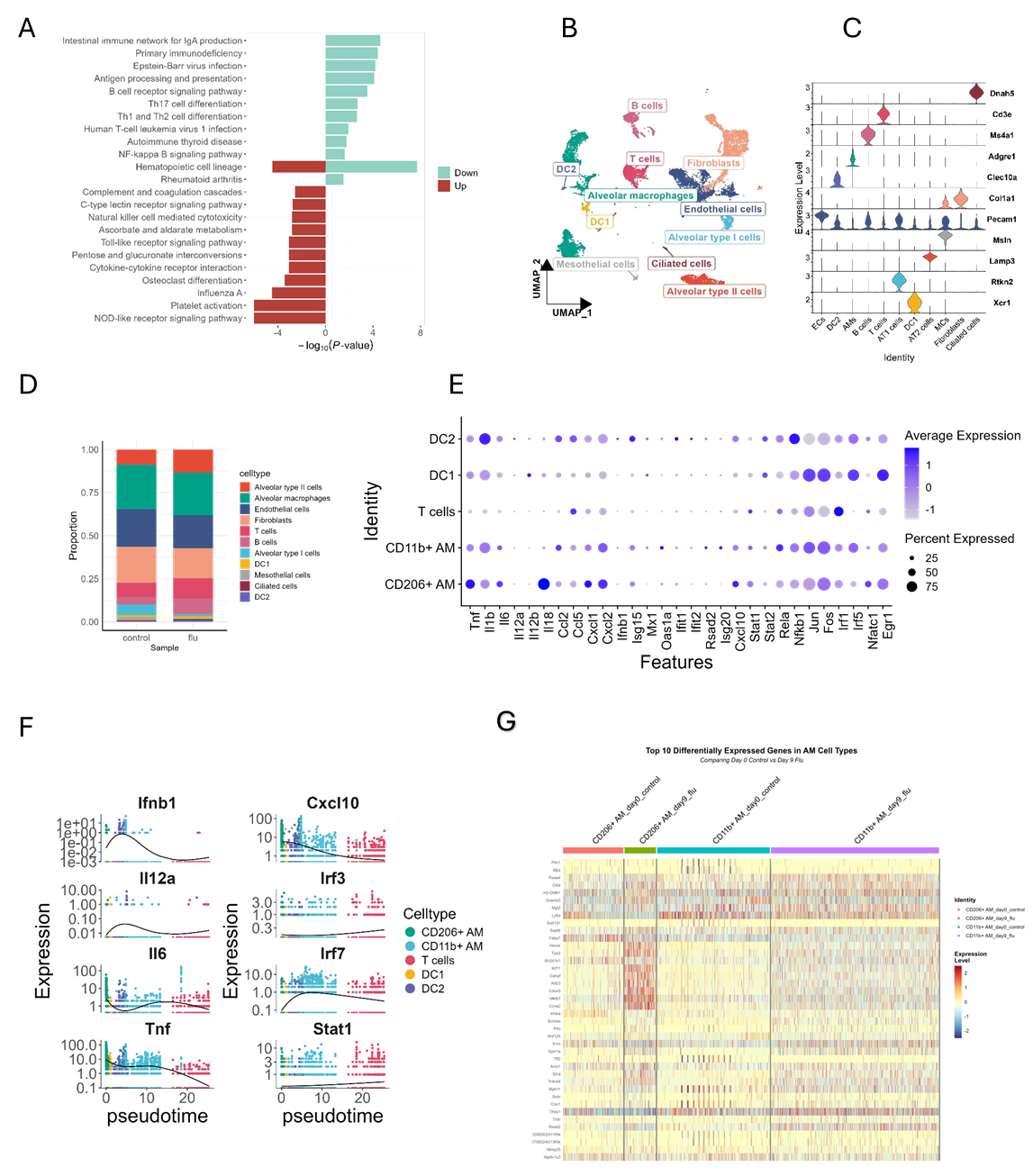


### **Figure S2 Transcriptomic Profiling Reveals Distinct IRF3/IRF7 Expression and Function in Macrophages versus Dendritic Cells During Influenza Infection.**

**(A)** KEGG pathway enrichment analysis of upregulated (red) and downregulated (blue) pathways in flu-infected mice. The pathways are ranked by -log10(p-value), with top pathways involved in immune response and inflammation highlighted. **(B-D):** Single-cell transcriptomics analysis of influenza-infected mouse lung tissue. (**B**): UMAP visualization of cell clusters.(**C**): Violin plots showing expression of marker genes across cell types. (**D**) Proportions of cell types in control and flu-infected samples. **(E)** Feature plot depicting gene expression profiles across immune cell populations. **(F**) the selected gene expression pattern in given cell types by Trajectory analysis. (**G**) Heatmap of differentially expressed genes between flu and control conditions


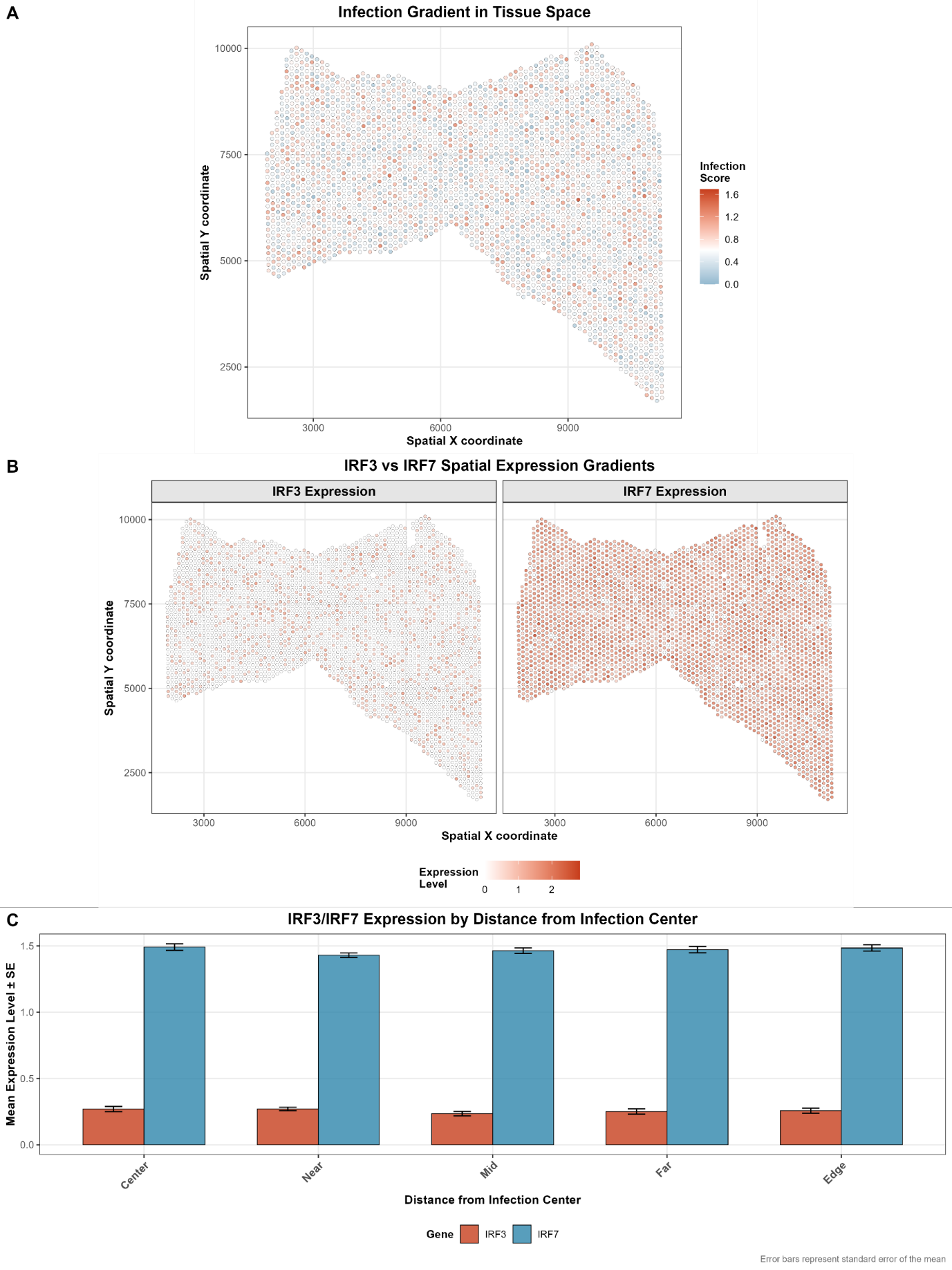


### **Figure S3 Spatial infection gradient analysis reveals heterogeneous IRF3 and IRF7 expression patterns in Day 9 post-influenza lung tissue.**

**(A)** Infection gradient heatmap showing the spatial distribution of infection scores across tissue coordinates. Infection scores were calculated as the mean expression of inflammation-associated genes (IRF3, IRF7, IFNB1, TNF, IL6, CXCL10). Each point represents a spatial transcriptomics spot, with colors indicating infection intensity from low (blue) to high (red). n = 2847 spots. Infection score range: 0.00 to 1.69. **(B)** Comparative spatial expression maps of IRF3 and IRF7. Left panel shows IRF3 expression levels across tissue space; right panel shows IRF7 expression. Expression levels are displayed as a gradient from low (blue) to high (red). IRF3 expression range: 0.00 to 1.79; IRF7 expression range: 0.00 to 2.83. **(C)** Distance-dependent expression analysis showing IRF3 and IRF7 expression levels as a function of distance from the infection center. The infection center was defined as the spot with the highest infection score. Spots were binned into five distance categories: Center, Near, Mid, Far, and Edge. Bar heights represent mean expression levels, with error bars indicating standard error of the mean (SEM). Statistical significance was assessed using Pearson correlation analysis.


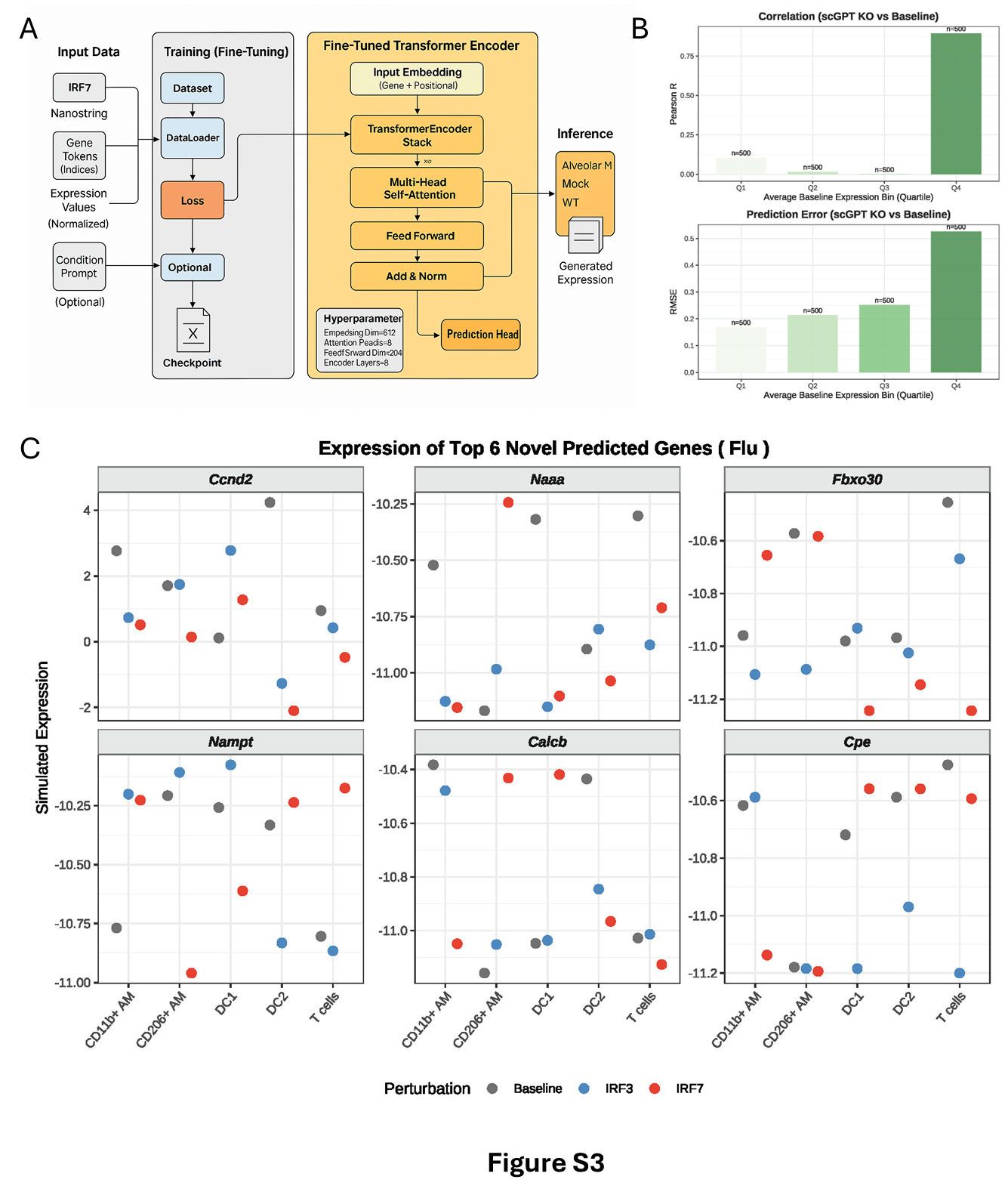


### **Figure S4 Schematic overview of the scGPT framework for conditional gene expression simulation in IRF3/IRF7 knockout and influenza-infected lung immune cells.**

**(A)** The schematic outlines the training and inference pipeline of the scGPT model applied to mouse single-cell RNA-seq data. Control condition expression profiles were used to construct the input feature space for training a 6-layer Transformer encoder (8 heads/layer; embedding dim = 512; hidden size = 2048) under a masked language modeling objective, with auxiliary supervision from cell type labels. For simulation, 512-dimensional condition prompts (e.g., AM-IRF7KO-IAV) were combined with gene tokens and random priors to generate condition-specific gene expression. Simulated outputs were compared against Nanostring, bulk RNA-seq, and scRNA-seq data to assess accuracy across key immune subsets, including alveolar macrophages (AM), DC1, and DC2.

**(B)** Simulated expression consistency and magnitude were evaluated across gene expression quartiles (Q1–Q4) based on baseline Flu condition. Top: Pearson correlation (R) between baseline and IRF3/IRF7 KO simulations. Bottom: Root Mean Square Error (RMSE) across the same bins. Both metrics increased sharply in the high-expression quartile (Q4), suggesting expression-dependent simulation fidelity. Each bin includes 500 genes.

**(C)** Simulated expression of six top-ranked genes (Ccnd2, Naaa, Fbxo30, Nampt, Calcb, Cpe) across three conditions (Baseline, IRF3 KO, IRF7 KO) and five cell types. Points are colored by condition and faceted by gene.


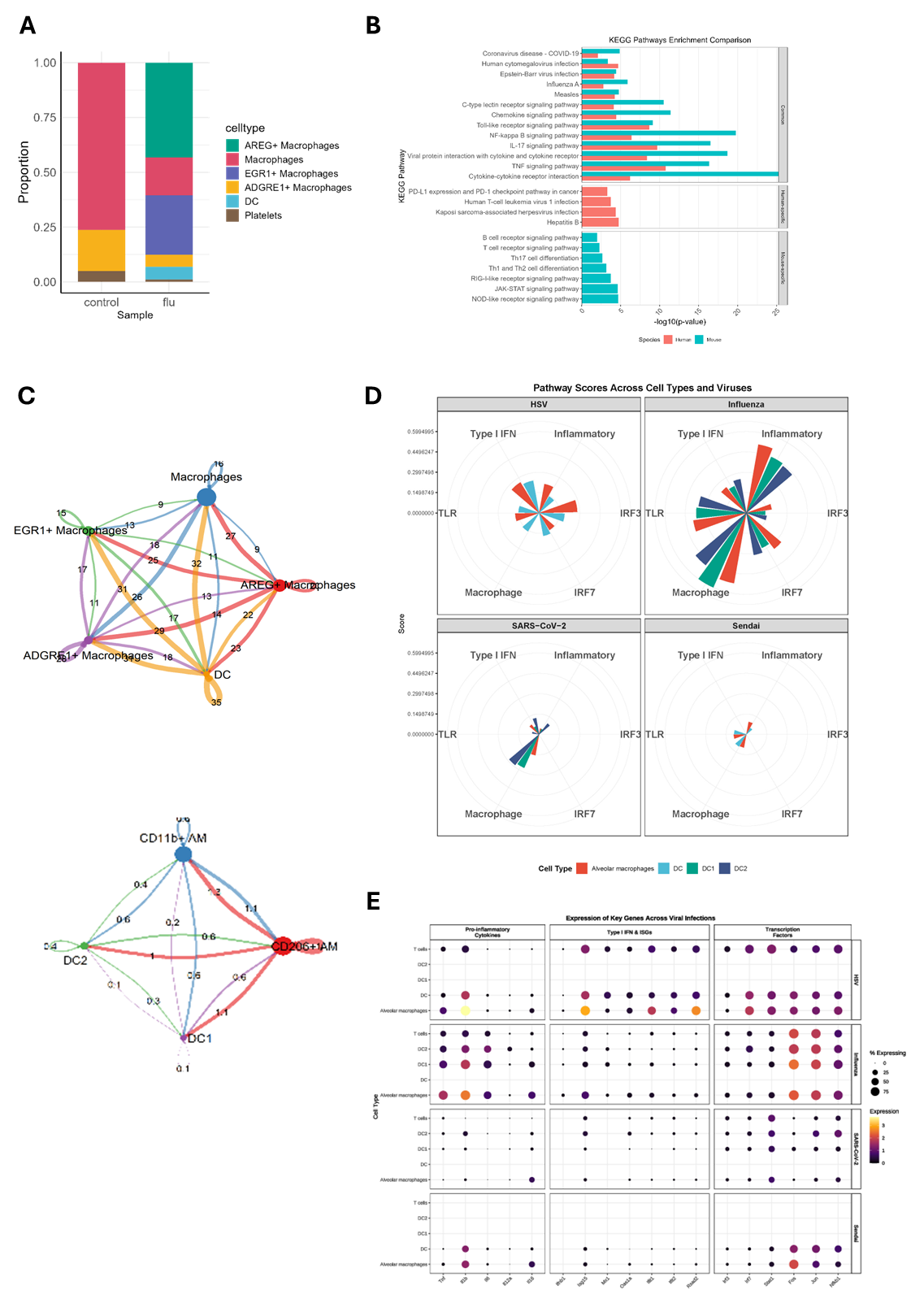


### **Figure S5 Cross-species and cross virus analysis reveals conserved and distinct irf3/irf7 functions in human and mouse macrophages and dendritic cells during different virus infection.**

1. Proportional bar graph displaying the relative abundance of identified immune cell types in control and influenza-infected human lung samples.
2. KEGG pathway enrichment comparison of upregulated genes between human and mouse post-influenza infection. The x-axis represents the -log10(p-value) of enriched pathways, while the y-axis lists the pathways. Red bars represent human data, and blue bars represent mouse data.
3. CellChat analysis showing intercellular communication strength for various immune cell types in humans (Top) and mice (bottom) post-influenza infection. The thickness of the lines corresponds to the strength of the interactions between the cell types.
4. Comprehensive Immune Pathway Activation Profiles Reveal Virus-Specific Response Patterns. Radar plots depicting the activation of key immune pathways in different cell types following viral infection. Each spoke represents a distinct immune pathway (Type I IFN, IRF3, IRF7, Inflammatory, TLR, and Macrophage activation pathways), and the distance from the center indicates the activation score. Cell types are shown in different colors, and plots are grouped by viral stimulus (HSV, Influenza, SARS-CoV-2, and Sendai virus). Values represent mean pathway scores derived from gene expression analysis. Values represent mean pathway scores derived from gene expression analysis of pathway-specific gene sets, with normalization to control conditions.
5. Dot plot depicting the expression of pro-inflammatory cytokines, type I interferon and ISGs, and transcription factors across cell types during viral infections.
